# Evolutionary history of two evergreen *Rhododendron* species as revealed by chromosome-level genome assembly

**DOI:** 10.3389/fpls.2023.1123707

**Published:** 2023-03-21

**Authors:** Xiaopei Wu, Lu Zhang, Xiuyun Wang, Rengang Zhang, Guihua Jin, Yanting Hu, Hong Yang, Zhenzhen Wu, Yongpeng Ma, Chengjun Zhang, Jihua Wang

**Affiliations:** ^1^ Germplasm Bank of Wild Species, Kunming Institute of Botany, Chinese Academy of Sciences, Kunming, China; ^2^ University of Chinese Academy of Sciences, Beijing, China; ^3^ Flower Research Institute of Yunnan Academy of Agricultural Sciences, National Engineering Research Center for Ornamental Horticulture, Kunming, China; ^4^ Genomics and Genetic Engineering Laboratory of Ornamental Plants, College of Agriculture and Biotechnology, Zhejiang University, Hangzhou, China; ^5^ Yunnan Key Laboratory for Integrative Conservation of Plant Species with Extremely Small Populations, Kunming Institute of Botany, Chinese Academy of Sciences, Kunming, China; ^6^ Key Laboratory for Plant Diversity and Biogeography of East Asia, Kunming, China; ^7^ State Key Laboratory of Biocatalysis and Enzyme Engineering, School of Life Sciences, Hubei University, Wuhan, China; ^8^ Zhejiang Institute of Advanced Technology, Haiyan Engineering & Technology Center, Jiaxing, China

**Keywords:** *Rhododendron delavayi*, *Rhododendron irroratum*, genome evolution, gene duplication, R2R3-MYB transcription factors, anthocyanin biosynthesis, NBS-encoding genes

## Abstract

**Background:**

The genus *Rhododendron* (Ericaceae), a species-rich and widely distributed genus of woody plants, is distinguished for the beautiful and diverse flowers. *Rhododendron delavayi* Franch. and *Rhododendron irroratum* Franch., are highly attractive species widely distributed in south-west China and abundant new varieties have been selected from their genetic resources.

**Methods:**

We constructed chromosome-scale genome assemblies for *Rhododendron delavayi* and *Rhododendron irroratum*. Phylogenetic and whole-genome duplication analyses were performed to elucidate the evolutionary history of *Rhododendron*. Further, different types of gene duplications were identified and their contributions to gene family expansion were investigated. Finally, comprehensive characterization and evolutionary analysis of R2R3-MYB and NBS-encoding genes were conducted to explore their evolutionary patterns.

**Results:**

The phylogenetic analysis classified *Rhododendron* species into two sister clades, ‘rhododendrons’ and ‘azaleas’. Whole-genome duplication (WGD) analysis unveiled only one WGD event that occurred in *Rhododendron* after the ancestral γ triplication. Gene duplication and gene family expansion analyses suggested that the younger tandem and proximal duplications contributed greatly to the expansion of gene families involved in secondary metabolite biosynthesis and stress response. The candidate R2R3-MYB genes likely regulating anthocyanin biosynthesis and stress tolerance in *Rhododendron* will facilitate the breeding for ornamental use. NBS-encoding genes had undergone significant expansion and experienced species-specific gain and loss events in *Rhododendron* plants.

**Conclusions:**

The reference genomes presented here will provide important genetic resources for molecular breeding and genetic improvement of plants in this economically important *Rhododendron* genus.

## Introduction

**Figure 1:**
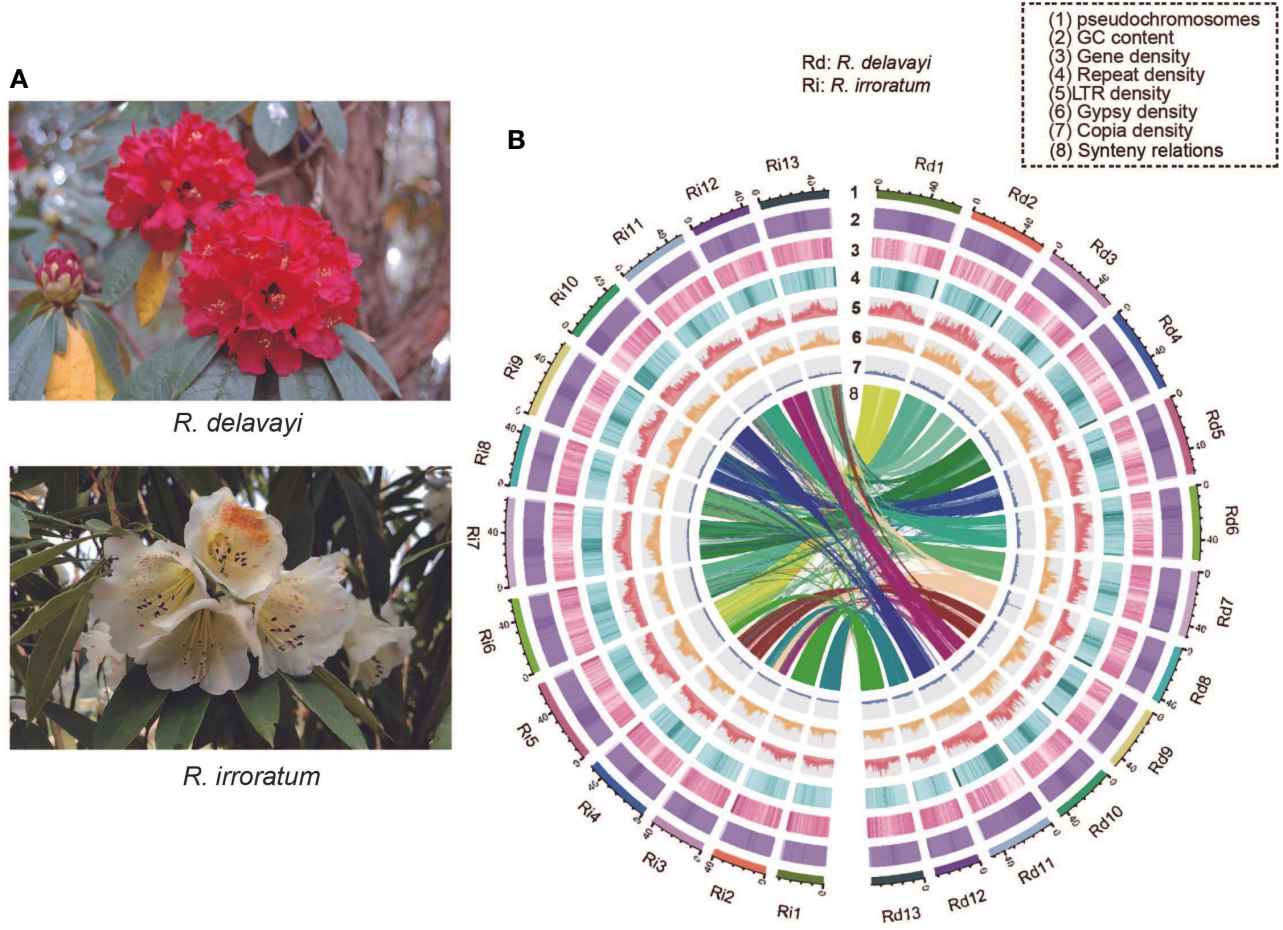
Flower morphology and genome features of *R. delavayi* and *R. irroratum*. **(A)** *R. delavayi* are characterized by bright red flowers and *R. irroratum* typically has pale yellow flowers. Photos: Hong Yang and Yan-Ting Hu, Baili Rhododendron Nature Reserve, Guizhou, China. **(B)** The landscape of the genome assemblies and annotations for *R. delavayi* and *R. irroratum.* The density was calculated in 100-kb sliding windows (Rd: *R. delavayi* and Ri: *R. irroratum*).

*Rhododendron* L., the largest genus in Ericaceae family, contains more than 1000 species and is widely distributed in Asia, Europe, and North America, with the center of diversity in southern China (Fang and Min, 1995; Chamberlain et al., 1996). To date, approximately 600 species have been confirmed from China, including many new species recognized in recent years (Tian et al., 2019). Because of their distinctive and colorful flowers, species within this genus are widely cultivated as horticultural plants with high ornamental, aesthetic and economic value. *Rhododendron delavayi* Franch. and *Rhododendron irroratum* Franch., are both evergreen and alpine species of *Rhododendron* subgenus *Hymenanthes* (Chamberlain, 1982) and widely distributed in south-west China. The *R. delavayi* has large, scarlet flowers and *R. irroratum* is often characterized by yellow corollas with yellow-green to lavender-red spots (**Figure 1**), which make them highly attractive and abundant new varieties have been selected from the *R. delavayi* and *R. irroratum* genetic resources.

As important ornamental plants, mining key candidate genes conferring flower coloration and stress tolerance are important for future breeding to develop new horticultural cultivars, and to enhance stress tolerance of *Rhododendron* cultivars to unfavorable environmental factors. The R2R3-MYB proteins accounted for the largest proportion of MYB superfamily in plants and are critical in controlling the biosynthesis of flavonoids/anthocyanin, which is the most important and widespread floral pigment (Tanaka et al., 2008; Wessinger and Rausher, 2012). Moreover, R2R3-MYB proteins are demonstrated to participant in responses to various stressful conditions, e. g., drought, cold and pathogen attack (Dubos et al., 2010). Nevertheless, the R2R3-MYB subfamily has not been well analyzed in *Rhododendron*. Thus, identifying and characterizing the *R2R3-MYB* genes in *Rhododendron* can provide reference for future functional verifications.

Through long periods of evolution, plants have evolved complicated defense mechanism to resist the invasion of various pathogens. The plant disease-resistance (R) genes play vital roles in recognition of virulence factors and inducing immune responses (Jones and Dangl, 2006; McDowell and Simon, 2006; Bent and Mackey, 2007). Nucleotide-binding site-leucine-rich repeat (*NBS-LRR*, NBS-encoding for short) genes make up the largest family (80%) of known plant R genes and are crucial in protecting plants against pathogenic organisms (Meyers et al., 1999; Dangl and Jones, 2001; Xiao et al., 2001; Meyers et al., 2003). The NBS-LRR proteins have been classified into three subclasses according to their phylogeny, that is, TIR-NBS-LRR(TNL), CC-NBS-LRR(CNL), and RPW8-NBS-LRR(RNL) (Shao et al., 2016; Shao et al., 2019). In recent years, *Rhododendron* flower rot, stem rot, root rot and leaf diseases are occurring with increasing frequently, causing serious losses of rhododendron plants and to local economies.

Up to date, several *Rhododendron* genomes have been released (**Supplementary Table 1**), including two draft rhododendron genomes (*R. delavayi* and *R. williamsianum*) obtained by Illumina short read sequencing (Zhang et al., 2017; Soza et al., 2019), two high-quality, chromosome-level azalea genomes (*R. simsii* and *R. ovatum*) generated by PacBio SMRT sequencing (Yang et al., 2020; Wang et al., 2021) and the recently sequenced high-quality genomes of *R. griersonianum* (Ma et al., 2021) and *R. henanense* (Zhou et al., 2022). These emerging genomic resources greatly promote the genomic research of *Rhododendron* and facilitate our understanding of the Ericaceae evolution. The available *R. delavayi* genome was generated using HiSeq Illumina sequencing, however the assembly was fragmented, which impedes the investigation of genome evolution and utilization of genetic resources. Therefore, the high-quality, chromosome-scale assembly for *R. delavayi* was constructed with Nanopore long read sequencing and Hi-C scaffolding in this study. Meanwhile, the *R. irroratum* was assembled to chromosome level by Combining PacBio SMRT (single-molecule real-time) and Hi-C sequencing. Phylogenetic analyses of *R. delavayi* and *R. irroratum* with other representative angiosperm species enabled us to further investigate the evolutionary relationships within the *Rhododendron* genus. Moreover, different patterns of gene duplications were searched and their contributions to gene family expansion were explored for the two sequenced genomes. Furthermore, the identification, comparative phylogenetic analysis and functional prediction of *R2R3-MYB* genes were performed in *Rhododendron* species. Finally, the systematic investigation of *Rhododendron* NBS-encoding genes were conducted to explore their chromosomal organization, evolutionary patterns and duplicated types of this gene family.

This study yielded insights into evolution history of the *Rhododendron* genus. It is hope that the comprehensive analysis of *R2R3-MYB* and NBS*-*encoding genes will provide basic and essential information for subsequent screening of the interesting flower coloration and disease resistance genes, which can facilitate genetic improvement of *Rhododendron* cultivars at molecular level.

## Materials and methods

### Plant materials and DNA sequencing

The plant materials of *R. delavayi* were collected from a 50-year-old tree growing in Jindian National Forest Park (Yunnan, China). This tree was transplanted from Cang Shan Mountain (Yunnan, China) in 1995. High-quality genomic DNA was isolated from fresh leaves with a QIAGEN^®^ Genomic Kit and then purified using a QIAquick Gel Extraction Kit. The DNA library was constructed using the SQK_LSK109 Ligation Sequencing Kit and sequenced on a PromethION System (Oxford Nanopore Technologies, UK) using one Nanopore cell. After base-calling (Guppy v4.0.1) and data quality control, a total of ~55 Gb Nanopore long reads were obtained with an average length and N50 size of 17 Kb and 23 Kb, respectively (**Supplementary Table 2**). The Hi-C library was prepared with Dpn II restriction enzyme following the method of previous studies (van Berkum et al., 2010) and sequenced on an Illumina HiSeq X-Ten machine, finally generating ~68  Gb clean paired-end (PE) reads.

Fresh leaf tissues of *R. irroratum* were collected from a wild plant in Baili Rhododendron Nature Reserve (Guizhou, China). The materials were immediately frozen in liquid nitrogen and stored at −80°C for DNA extraction and library construction. The CTAB method was adopted to extract the genomic DNA. For short read sequencing, the short paired-end library (400 bp) was created and sequenced on the MGISEQ-2000 platform (PE100). Raw sequencing data was processed with fastp v0.20.1 (Chen et al., 2018) to obtain ~23 Gb clean data. For PacBio SMRT sequencing, the SMRTbell libraries with 20 Kb inserts were prepared and sequenced on the PacBio Sequel sequencer with 17 SMRT cells (Pacific Biosciences, CA, USA) using DNA Sequencing Reagent Kit 4.0 v2. After data filtering and preprocessing, ~71 Gb clean data were obtained, which had a mean subread length and N50 size of 7,681 bp and 12,150 bp, respectively (**Supplementary Table 2**). The method of Hi-C library preparation for *R. irroratum* was the same to *delavayi* using Dpn II restriction enzyme and ~74 Gb clean data were generated after sequencing on an Illumina Hiseq X-Ten platform.

### Transcriptome sequencing and preprocessing

To support the gene annotation of *R. irroratum*, leaf and flower bud tissues were collected to prepare RNA samples and ~1 μg RNA were used to construct the sequencing libraries with NEBNext^®^Ultra™ RNA Library Prep Kit for Illumina^®^(NEB, USA) by following the manufacturer’s instructions. The libraries were sequenced on the Illumina HiSeq 2000 platform (PE150) and ~42 Gb clean data were obtained (**Supplementary Table 3**). In addition, leaf and flower bud tissues from *R. irroratum* were mixed to perform PacBio Iso-Seq sequencing. The PacBio Iso-Seq3 pipeline^1^ was applied to preprocess raw reads to generate high-quality, full-length and consistent isoform transcripts, finally producing 21,499 transcripts (**Supplementary Table 3**).

### Estimation of genome size and heterozygosity

The genome size and heterozygosity for *R. delavayi* and *R. irroratum* were evaluated by *K*-mer analysis. In addition, flow cytometry measurement was also conducted to estimate the size of *R. irroratum* genome. Young leaves of *R. irroratum* were sampled at Kunming Botanical Garden (Yunnan, China) and nuclear DNA was isolated using a customed protocol. Then, nuclear DNA content was measured by flow cytometry using *Zea mays* ‘B73’ as reference standard. For *K*-mer analysis, Jellyfish v2.2.10 (Marcais and Kingsford, 2011) were applied to calculate *K*-mer counts and GenomeScope v1.0 (Vurture et al., 2017) estimated the genome size, repeat content, and heterozygosity.

### Genome assembly and quality assessment

The Nanopore reads were applied to assemble contig sequences of *R. delavayi*. First, NextDenovo^2^ was used to correct errors in the reads. Then, SMARTdenovo (Liu et al., 2020a), WTDBG^3^, and NextDenovo were applied to assemble the corrected long reads, respectively. The three softwares yielded the assemblies of 765, 790 and 690 Mb, with contig N50 of 1,900, 500, and 11,000 Kb, respectively (**Supplementary Table 4**). Finally, the preassembly generated by NextDenovo was regarded as the optimal assembly for further analysis. Pilon (Walker et al., 2014) was applied to correct the contigs with pair-end short reads (~100 Gb) sequenced in the previous research (Zhang et al., 2017). After that, Juicer (Durand et al., 2016b) and 3D-DNA pipeline (Dudchenko et al., 2017) were used to anchor contig sequences into chromosome-scale scaffolds using Hi-C data. Boundaries of these scaffolds were manually adjusted in Juicebox (Durand et al., 2016a) and each scaffold was re-scaffolded in 3D-DNA, followed by manual adjustment within Juicebox to fix insert, bound, order, and mis-join errors. LR_Gapcloser (Xu et al., 2019) was used to close gaps twice by mapping the ONT long reads to Hi-C assembly. Subsequently, three rounds of genome polishing were conducted by NextPolish (Hu et al., 2020) with pair-end short reads. The heterozygous and redundant sequences in scattered contigs (contigs which were not anchored into chromosomes) were removed by Redundans (Pryszcz and Gabaldón, 2016). Finally, the *R. delavayi* genome sequences contained 13 pseudo-chromosomes, and 10 unplaced contigs. Three strategies were employed to assess the quality of final assembly. Firstly, the accuracy was evaluated by aligning Illumina and ONT reads to final genome using BWA-MEM (Li and Durbin, 2009) and minimap2 (Li, 2018), respectively. Next, BUSCO analysis (version 5.4.5, eudicots_odb10) was adopted to evaluate the completeness of the assembly. Finally, the continuity of assembly was evaluated by LAI index (Ou et al., 2018).

The *R. irroratum* genome sequence was assembled by integrating the PacBio, Hi-C and MGI-SEQ sequencing. To obtain an optimal primary assembly, SMARTdenovo, WTDBG, Canu (Koren et al., 2017) and FALCON/FALCON-Unzip (Chin et al., 2016) were initially used to assemble the PacBio subreads, producing four versions of primary assemblies (**Supplementary Table 4**). The assembly generated by FALCON and FALCON-Unzip was selected as the optimal assembly with the maximum N50 and fewest contigs. Subsequently, duplicated assembled haploid contigs were removed by Purge Haplotigs (Roach et al., 2018), reducing the assembled size from 853.63 to 700.47 Mb. The obtained curated contigs were scaffolded using the SSPACE-longread v1.1 (Boetzer and Pirovano, 2014). Then, ALLHiC pipeline (Zhang et al., 2019) anchored the scaffolds into super-scaffolds, which were subjected to inspection and manually adjusted in Juicebox v1.11.08 to generate chromosome-level scaffolds. Finally, the genome sequence was polished by Pilon with paired-end short reads. In result, the *R. irroratum* assembly was composed of 13 pseudo-chromosomes and 1440 unanchored contigs. Three methods used for quality control of the *R. delavayi* genome were also applied to evaluate the quality of *R. irroratum* assembly. PacBio subreads, pair-end short reads and full-length transcripts were mapped to final assembled sequences.

### Genome annotation

Genome annotation of *R. delavayi* can be divided into two parts: annotation of repeat elements and annotation of gene structure and function. We employed a combined method of homology-based search and *ab initio* identification to annotate the repeat elements. For *ab initio* method, LTR_retriever (Ou and Jiang, 2018) and RepeatModeler v1.0.11 were used to find repeat elements, which were merged as the *ab initio* repeat library to identify repetitive sequences by RepeatMasker (Chen, 2004). The homology-based strategy was performed by searching a known repeat library (Repbase 20170127) with RepeatMasker. We combined repetitive sequences predicted by both methods and regarded them as the final repeat set. Then, the identified repeat elements were masked using RepeatMasker and gene models were predicted by MAKER2 pipeline (Holt and Yandell, 2011). The 83,515 transcripts from the mixed sample (containing five different tissues: flowers, flower buds, young leaves, mature leaves, and young stems) and 187,124 combined non-redundant protein sequences from *R. delavayi*, *R. simsii*, *R. williamsianum*, and *Vaccinium corymbosum*, *Actinidia chinensis*, and *Arabidopsis thaliana* were considered as evidence of ESTs and homolog proteins, respectively.

The procedure of genome annotation for *R. irroratum* was similar to that of *R. delavayi*. Firstly, we identified the repeat elements by combining evidence from homology alignments and *ab initio* prediction. The homology-based approach was the same with *R. delavayi*. LTRharvest, LTR_FINDER (Xu and Wang, 2007) and RepeatModeler were applied to construct the *ab initio* repeat library. Then, the repeat database from the above two strategies was used to mask the genome using RepeatMasker. To perform gene structure predictions, the Illumina RNA-seq reads and high-quality full-length transcripts generated by Iso-Seq sequencing were aligned to the repeat-softmasked genome using STAR (Dobin et al., 2013) and GMAP (Wu and Watanabe, 2005), respectively. Proteins of *R. williamsianum*, *Solanum lycopersicum*, *Camellia sinensis*, *R. delavayi*, *A. chinensis* and *V. corymbosum* were selected for homologous protein alignment. The BRAKER2 pipeline v2.1.5 (Hoff et al., 2019), which combines GeneMark-ES/ET and AUGUSTUS, was applied to annotate gene models by integrating evidence from RNA-seq, Iso-Seq and homologous proteins. In addition, we also utilized the GeMoMa (Keilwagen et al., 2016; Keilwagen et al., 2018) to perform homology-based prediction by using genome sequences and gene models of *R. williamsianum*, *Solanum lycopersicum*, *Camellia sinensis*, *R. delavayi*, *A. chinensis* and *V. corymbosum*. Moreover, we employed RNA-seq and Iso-Seq data to conduct further RNA-seq-based prediction. Trinity v2.8.5 (Haas et al., 2013) was used to assemble the Illumina RNA-seq reads into transcripts. The obtained transcripts combined with full-length transcripts were aligned to final assembly by PASA (Campbell et al., 2006). Meanwhile, the candidate coding regions in the transcripts were identified by TransDecoder^4^. Finally, the consensus gene set was generated by combining the gene models predicted by different strategies with EVidenceModeler v1.1.1 (Haas et al., 2008)

Protein functional annotations were carried out by BLASTP (E-value: 1e−5) (Altschul et al., 1990) homology searches against the NCBI non-redundant protein database (2020-7-10) and Swiss-Prot database (2020-8-12). InterProScan package (version 5.29-68.0) was used to perform a comprehensive annotation such as Pfam, GO and KO. EggNOG-mapper v1.0.3 was used for functional annotation based on the eggNOG database. GO and KO information was retrieved from InterPro and eggNOG annotation.

### Gene family, comparative genomics and evolutionary analysis

We selected 19 representative angiosperms (including *R. delavayi* and *R. irroratum*) (**Supplementary Table 5**) to identify orthogroups (orthologous gene families) by using the OrthoMCL pipeline^5^ (parameter: min_length: 50, inflation: 1.5, https://github.com/apetkau/orthomcl-pipeline), which used the all-versus-all BLASTP (E-value: 1e-5) results of all proteins. The single-copy orthogroups (orthogroups that contained one only gene for each species) were selected for phylogenetic analysis with concatenated approach. First, the proteins of each single-copy orthogroup were aligned by MAFFT v3.8.1551 (Katoh and Standley, 2013) and the protein alignments were converted into corresponding coding sequence (CDS) alignments using PAL2NAL v14 (Suyama et al., 2006), followed by refinement with trimAI v1.480 (Capella-Gutierrez et al., 2009). Then, CDS alignments were concatenated to a super gene matrix. jModelTest v2.1.7 (Posada, 2008) was applied to select the optimal substitution model. Finally, a maximum likelihood (ML) tree was constructed using RAxML v8.2.12 (Stamatakis, 2014) with the concatenated CDS alignments based on GTR+I+GAMMA model (1000 bootstrap tests). Orthogroups expansion and contraction for each species were inferred by CAFE v4.2.1 (De Bie et al., 2006). Orthogroups were filtered out when one or more species contains more than 100 gene copies. Divergence times among the 19 species were estimated using MCMCtree implemented in PAML package (Yang, 1997) with ‘JC69’ model and parameters ‘burn-in = 10,000,000, nsample = 200,000, and sample-frequency = 5,000’. Two fixed fossil ages: (1) Ericales (89.8 Mya) (Nixon and Crepet, 1993), (2) *Rhododendron* (56 Mya) (Collinson and Crane, 1978) and five soft-bound calibration points acquired from document (Eudicots: 125–161 Mya, Angiosperms: 199–167 Mya and Asterids–Rosid: 116–126 Mya) (Li et al., 2019), and TimeTree website (*A. comosus*–*O. sativa*: 102–120 Mya and *A. thaliana*–*V. vinifera*: 107–135 Mya) to were used to calibrate the age of the nodes in the tree.

### Whole-genome duplication analysis

Three approaches were adopted to elucidate the WGDs in *Rhododendron*. First and foremost is the synonymous (*Ks*) substitution rates distribution. Collinear blocks (more than five collinear genes) within and between species were identified by MCScanX (Wang et al., 2012) based on the BLASTP (E-value: 1e-5) results of proteins. The protein-coding DNA alignments for collinear gene pairs were performed using ParaAT v2.0 (Zhang et al., 2012). *Ks* values of the collinear gene pairs were calculated by KaKs_Calculator v2.0 (Wang et al., 2010) with the YN model. Then, the microsynteny of grape and *R. delavayi* and of grape and kiwifruit were depicted by MCScan Python version^6^. Moreover, to explore whether kiwifruit and *Rhododendron* shared the same Ad-β WGD or experienced lineage-specific WGDs shortly after their divergence, we extracted collinear gene pairs falling in the *Ks* peak ± 1 s.d. as paralogous pairs that likely derived from Ad-β event in *R. delavayi* and kiwifruit, respectively, referring to the methods described by Teh (Teh et al., 2017). Next, gene family clustering was performed using proteins of kiwifruit, *R. delavayi* and *C. canephora* with the OrthoMCL pipeline and orthogroups that contained one such paralogous pair of *R. delavayi*, one such paralogous pair of kiwifruit, and one gene from *C. canephora* were extracted. In each of such orthogroups, each *R. delavayi* paralog was matched with its kiwifruit ortholog (among the two paralogs), by setting the *R. delavayi*–kiwifruit pair with the lowest synonymous distance as orthologs. RAxML constructed the phylogenetic relationships of the paralogs and orthologs in *R. delavayi* and kiwifruit, along the genes from *C. canephora*, which experienced no additional WGDs expect the ancient WGT-γ.

The time of β WGD event in *Rhododendron* was estimated according to the formula T = *Ks*/2r, where r is synonymous substitutions at each site per year, which was calculated according to the mean *Ks* peaks of BLASTP reciprocal best hit (RBH) pairwise sequences of kiwifruit vs. five *Rhododendron* species (*R. delavayi*, *R. williamsianum*, *R. irroratum*, *R. simsii* and *R. ovatum)* and kiwifruit - *Rhododendron* divergence time (median: 71 Mya) as an age constraint, following r=*Ks*/(2 × (divergence time)).

### Analysis of gene duplication

To further understand the gene duplication and evolution in *R. delavayi* and *R. irroratum* genomes, we determined different classes of gene duplications with *DupGen_finder* pipeline (Qiao et al., 2019), which categorized the duplicated genes into 5 types: whole-genome duplicates, tandem duplicates (adjacent gene copies in the same chromosome), proximal duplicates (gene pairs located on nearby chromosomal region but separated by less than 10 genes), transposed duplicates (gene pairs originating from DNA-based transposition or retrotransposition) and dispersed duplicates (other types excluding the above mentioned). The *Ks* values for each type of duplicated pairs were computed by KaKsCalculator v2.0.

### Identification and characterization of R2R3-MYB subfamily transcription factors

The R2R3-MYB subfamily members belonging to MYB superfamily were identified in four *Rhododendron* species (*R. delavayi*, *R. irroratum*, *R. ovatum*, and *R. simsii*) investigated in this study. *R. williamsianum* was excluded from the analysis due to the poor quality of its genome sequence. The Hidden Markov Model (HMM) profile of MYB DNA-binding domain (PF000249) was downloaded from the Pfam website^7^ and used as a query to search the *Rhododendron* protein sequences using HMMER v3.1b2 (E-value: 1e-5). In addition, PlantRegMap^8^ was also used to predict transcription factors with best hits in *A. thaliana*. The obtained MYB protein sequences were merged and redundant sequences were removed. Then, proteins that did not have the homologous SwissProt annotation (E-value: 1e-5) or included incorrect domains depending on TAPscan v.2 transcription factor database were filtered. The obtained protein sequences were examined by SMART^9^, Pfam, and CD search^10^ analyses to validate the existence of MYB domain. Finally, the candidate sequences that contained two DNA-binding repeats were categorized as the R2R3-MYB subfamily. The MEME online tool was used to analyze conserved motifs of R2R3-MYB proteins (Bailey et al., 2006) with the following parameter settings: maximum number of different motifs, 20; minimum motif width, 6; and maximum motif width, 50. The exon/intron organizations of *R2R3-MYB* genes were visualized by TBtools v1.098696 (Chen et al., 2020). Chromosomal distributions of *Rhododendron R2R3-MYB* genes were retrieved from genome annotation files.

To explore the subgroup classification and phylogenetic relationships of *R2R3-MYB* proteins in *Rhododendron*, 125 R2R3-MYB protein sequences from *Arabidopsis* were retrieved from the Arabidopsis Information Resource^11^ (TAIR) and these proteins together with R2R3-MYB proteins from four *Rhododendron* species were aligned by MAFFT and a ML phylogenetic tree was constructed using IQ-TREE v1.6.12 (Nguyen et al., 2015) with 1000 bootstrap tests. The classification and categorization of *Arabidopsis R2R3-MYB* genes assisted the subdivision of *Rhododendron R2R3-MYB* genes. BLASTP (E-value: 1e-10) and MCScanX were applied to determine intraspecific synteny of the *Rhododendron R2R3-MYB* genes. TBtools plotted the 1:1 collinearity genes among four *Rhododendron* species.

The contributions of different gene duplications to *R2R3-MYB* subfamily gene expansion in *Rhododendron* species were further investigated by identifying the duplicated types of R2R3-MYB genes using *DupGen_finder*.

### Identification and evolutionary analyses of NBS-encoding genes

The HMM searches were also conducted to identify candidate NBS-encoding genes in kiwifruit and four *Rhododendron* plants. The HMM profile of NB-ARC domain (PF00931) was downloaded from Pfam website and used to search the protein sequences of kiwifruit and four *Rhododendron* species with HMMER v 3.1b2. The high quality sequences (E-value: 1e-20) obtained were used for multiple alignment with ClustalW (Thompson et al., 1994) and species-specific NBS profiles were constructed using HMMER software with the “hmmbuild” module. Using the species-specific profiles, the candidate NBS-encoding genes were obtained for each species and then submitted to online Pfam database to further confirm the presence of NB-ARC domain. All candidate NBS-encoding genes were further inspected using Pfam, SMART, CD search to examine whether they had TIR, RPW8, or LRR domains. Moreover, COILS program (Lupas et al., 1991) identified the CC motifs using the threshold of 0.9. Chromosomal locations of the NBS-encoding genes were retrieved from genome annotation files and cluster assignment of NBS-encoding genes followed the criterion used for *Medicago truncatula* (Ameline-Torregrosa et al., 2008): if the interval of two adjacent genes on the same chromosome was less than 250 Kb, these two genes were considered to be in the same cluster. According to this standard, the NBS-encoding genes were assigned to singletons and clustered loci.

The amino acid sequences of NB-ARC domain were extracted from the NBS-encoding genes of four *Rhododendron* species and kiwifruit and subjected to multiple sequence alignment with ClustalW integrated in MEGA-X (Kumar et al., 2018). After removing too short and exceedingly divergent sequences from the alignment, the ML NBS-encoding gene phylogeny was constructed using IQ-TREE with bootstraps of 1000 replicates. To understand the evolutionary history of this gene family, two phylogenies (CNL and nCNL (RNL and TNL)) were reconstructed for four *Rhododendron* species and kiwifruit. Gene duplication and loss events in the CNL and nCNL phylogenetic trees were restored by reconciling these two gene trees with real species tree through Notung software v2.9 (Stolzer et al., 2012). On the reconciled trees, if the NBS-encoding genes constituted a monophyletic clade that originated from the common ancestor of four *Rhododendron* species, then this clade was defined as a *Rhododendron* NBS-encoding lineage. If a monophyletic NBS-encoding gene branch originated in the common ancestor of *Rhododendron* and kiwifruit, this branch was defined as an Ericales NBS-encoding gene lineage. The duplications and losses of NBS-encoding genes during different evolutionary periods were restored at each node of the phylogenetic trees.

The roles of different gene duplications in expanding the *Rhododendron* NBS-encoding genes were also investigated following the method described above.

### Functional enrichment analysis

GO (Gene Ontology) and KEGG (Kyoto Encyclopedia of Genes and Genomes) enrichment analyses were performed using R package clusterProfiler (Yu et al., 2012) by setting all annotated *R. delavayi* or *R. irroratum* genes as background gene set. We used the corrected *P-*value (q value) < 0.01 as threshold criteria to judge whether a specific GO term or KEGG pathway was significantly overrepresented.

## Results

### Genome assembly and annotation

The genome size of *R. delavayi* and *R. irroratum* were estimated to be ~647 Mb and ~597 Mb by *K*-mer analysis, respectively (**Supplementary Figure S1**). However, flow cytometry analysis estimated the haploid genome size of *R. irroratum* to be 730 Mb, which was higher than the *K*-mer method for yet unknown reasons (**Supplementary Figure S1**). Compared to *R. delavayi*, *R. irroratum* demonstrated a relatively high heterogeneity rate (**Table 1**; **Supplementary Table 1**). Here, ~ 55 Gb of Nanopore long reads and ~ 71 Gb of PacBio subreads were generated for *R. delavayi* and *R. irroratum*, respectively. Several assemblers were employed to conduct initial assembly and the optimal primary assemblies with a contig N50 of 11 Mb for *R. delavayi* and of 0.60 Mb for *R. irroratum*, were used for subsequent improvement (**Supplementary Table 4**). Through Hi-C scaffolding, 99.56% (659.80 Mb) of the sequences for *R. delavayi* and 92.96% (652.29 Mb) for *R. irroratum* were anchored, ordered and oriented into 13 pseudochromosomes (**Table 1**; **Supplementary Figure S2**). Three strategies were adopted to evaluate the quality of the assemblies. The accuracy of the assemblies were evidenced by the high genome coverage (> 98% for both *R. delavayi* and *R. irroratum*) and mapping rates (> 90% for both *R. delavay* and *R. irroratum*) (**Supplementary Table 6**). BUSCO analysis revealed that 2,264 (97.4%) and 2,251 (96.8%) of the 2,326 conserved single-copy orthologs of eudicots were completely recovered in the *R. delavayi* and *R. irroratum* genomes, respectively (**Supplementary Table 7**), indicating the completeness of the assemblies. Furthermore, high LTR Assembly Index (LAI) scores of 11.17 and 13.72 were estimated for *R. delavay* and *R. irroratum*, respectively, suggesting that the two newly assembled genomes reached the reference level.

**Table 1:** Genome survey, nuclear genome assembly and annotation features of *R. delavayi* and *R. irroratum*.

Feature	*R. delavayi* ^17^	*R. delavayi* (this study)	*R. irroratum* (this study)
Genome survey			
Estimate genome size (Mb)	∼697–717	647 (*K-*mer)	597 (*K-*mer)
Repeat content	–	65.6%	62.75%
Heterozygosity	0.9–1.1%	~1.1%	~1.55%
Genome assembly			
Assembly size (Mb)	695	662. 80	701.62
Contig No.	209, 969	216	1,549
Contig N50 (bp)	61, 801	11,000,000	695,292
Scaffold No.	193,091	23	1,413
Scaffold N50 (bp)	637,826	54,547,292	51,021,190
Chromosome-scale length (bp)	–	659,801,287 (99.56%)	652,297,987 (92.96%)
Complete BUSCOs	92.80%	97.4%	96.8%
LAI index	–	11.17	13.72
Genome annotation			
Repeat content	51.77%	51.00%	47.71%
No. of predicted protein coding genes	32,938	40,671	49,421
No. of functionally annotated (%)	85.91%	92.19%	88.92%
Complete BUSCOs	87.4%	90.9%	88.9%

Short line (-) indicated that data were not shown in the original article. Contig No. and Contig N50 were calculated from the optimal primary (contig-level) assembly. Scaffold No. and Scaffold N50 were calculated from the final chromosome-scale assembly. The assembly and annotation only considered the nuclear genome.

By combining the evidence from homology searches and *ab initio* prediction, 51.04% (338.93 Mb) and 47.71% (334.77 Mb) repeat elements were annotated in *R. delavayi* and *R. irroratum*, respectively (**Supplementary Table 8**). Long terminal repeat retrotransposons (LTR-RTs) are the predominant type of repeat elements and occupied 38.15% (253.29 Mb) of the *R. delavayi* and 32.78% (229.97 Mb) of the *R. irroratum* genome. DNA transposons accounted for 2.45% (16.29 Mb) of the *R. delavayi* and 2.00% (14.06 Mb) of the *R. irroratum* genome. The protein coding gene models were annotated using both evidence-based and *ab initio* prediction. In total, 40,671 and 49,421 coding genes were predicted for *R. delavayi* and *R. irroratum*, respectively. The annotated gene number of the current *R. delavayi* genome exceeded that of previous genome obtained by Illumina short read sequencing (32,938). The predicted genes were characterized by a mean length of 4,707 bp for *R. delavayi* and of 4,323 bp for *R. irroratum*, and an average CDS length of 1,195 bp for *R. delavayi* and 1,096 bp for *R. irroratum* (**Supplementary Table 9**). Of the annotated coding genes, 37,586 (92.19%) in *R. delavayi* and 43,946 (88.92%) in *R. irroratum* could be functionally annotated (**Supplementary Table 10**). Additionally, 99.69% of the *R. delavayi* and 93.96% of the *R. irroratum* genes could be assigned to the 13 pseudo-chromosomes. The annotation quality was also estimated based on the BUSCO analysis and 90.9% complete BUSCOs in *R. delavayi* and 88.9% in *R. irroratum* were detected, respectively (**Supplementary Table 11**). The GC content, gene density, repeat density, LTR density, Gypsy density and Copia density throughout the genome were given in **Figure 1B**.

### Phylogenetics and genome evolution

**Figure 2:**
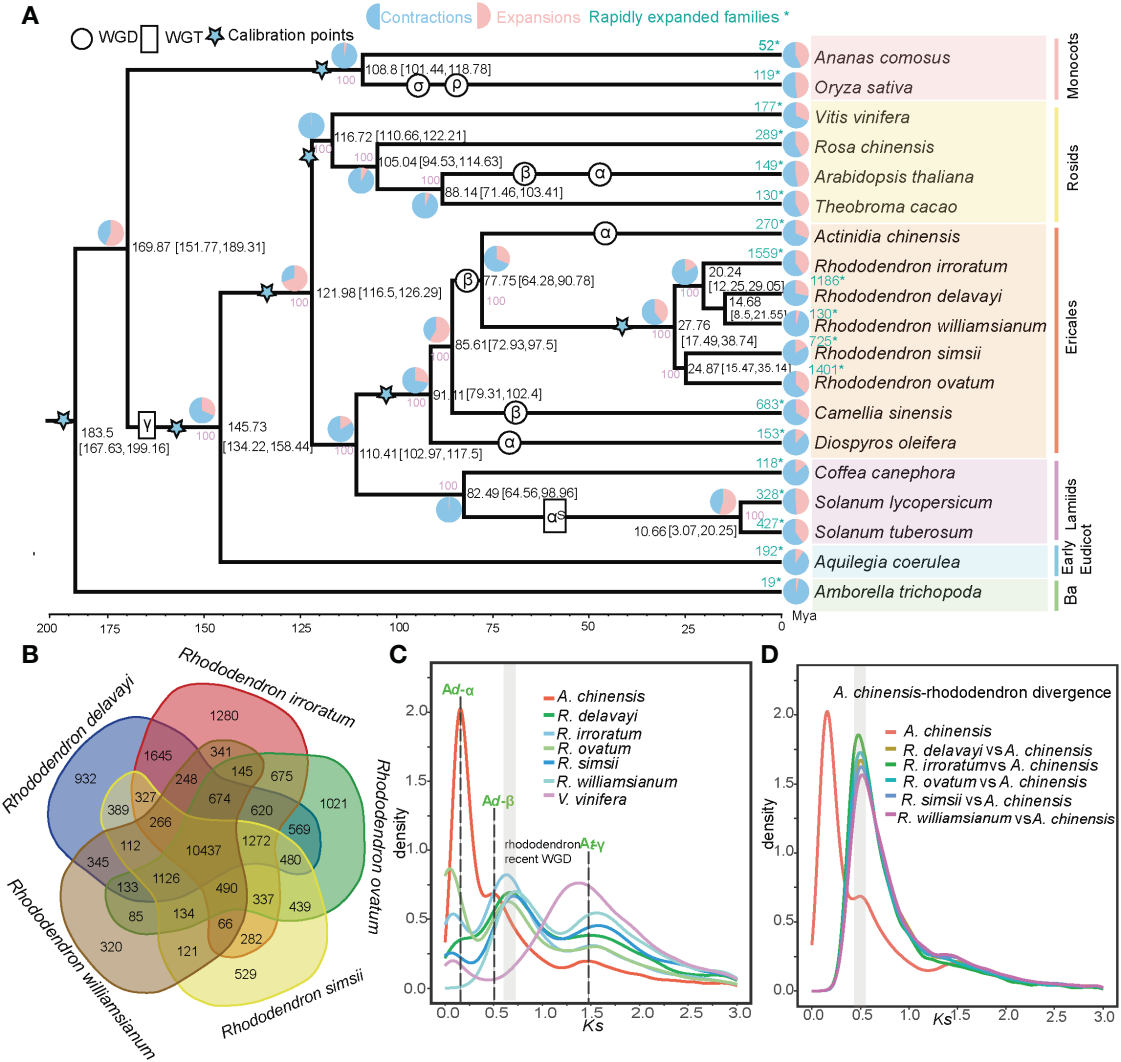
Evolutionary and comparative genomic analyses of *Rhododendron* species and representative angiosperms. **(A)** The phylogenetic tree of 19 angiosperms. All branches had 100% bootstrap support. Divergence time is displayed with 95% HPD in parentheses. Blue stars represented the calibration points. Estimated WGD/WGT events were placed according to results from our study and from those of previous investigations. The pink, light blue and light green numbers along particular branches indicated the number of orthogroups that undergone expansion, contraction and rapid evolution, respectively, * indicated the rapidly expanded families. **(B)** Shared and unique orthogroups among five *Rhododendron* species. **(C)** Synonymous substitution rate (*Ks*) distributions of syntenic paralogous genes for five.Rhododendron species, *V. vinifera* and *A chinensis* Ad-α; Ad-β and At-γ are illustrated with dotted lines. **(D)** *Ks* distributions of syntenic orthologous genes between *A chinensis* and five *Rhododendron* species.

To elucidate genome evolutionary history in *Rhododendron* species, gene family (orthogroup) clustering were carried out using proteins of *R. delavayi*, *R. irroratum* and 17 other sequenced angiosperms. Totally, 46,033 orthogroups were identified, of which 5,213, 7,954, 10,437 orthogroups were shared by all investigated angiosperms, Ericales and *Rhododendron*, respectively (**Supplementary Figure S3**; **Supplementary Table 12**). The 10,437 *Rhododendron*-shared gene families can considerably signify the “core” proteomes of *Rhododendron* species (**Figure 2B**) and they were enriched in functional categories termed “biosynthetic, metabolic and catabolic process”, and “response to fungus/bacterium/nematode” involved in metabolism and stress response based on GO enrichment analyses (**Supplementary Figure S4**). A phylogenetic tree was inferred for the 19 selected angiosperms, using 253 single-copy gene families (**Figure 2A**). The phylogenetic placement of main lineages agreed with previous studies (The Angiosperm Phylogeny Group et al., 2016). The *Rhododendron* genus was further divided into two clades: one clade comprising *R. delavayi*, *R. irroratum* and *R. williamsianum* (the evergreen rhododendrons) and the other containing *R. simsii* and *R. ovatum* (the azaleas). Based on calibration points, *Rhododendron* diverged from *Actinidia* (kiwifruit) at around 77.75 million years ago (Mya) and the divergence time of the two *Rhododendron* clades were estimated at around 27.76 Mya (**Figure 2A**). A total of 6,005 expanded genes in *R. delavayi* and 8,717 in *R. irroratum* belonged to 1,186 and 1,559 rapidly expanded gene families (EGFs), respectively (**Supplementary Table 13**, **Figure 2A**). KEGG enrichment analyses suggested the rapidly EGFs of two sequenced species in this study were both highly enriched in “plant-pathogen interaction”, “cutin, suberine and wax biosynthesis”, “terpenoid biosynthesis” and “anthocyanin biosynthesis” pathways (**Supplementary Figure S5**). Besides, GO enrichment for the rapidly EGFs revealed that there was a remarkable enrichment in GO terms involved in biotic stimuli and defense responses in the two species, for example, “cellular response to biotic stimulus”, “defense response to insect/fungus”, “immune response-activating signal transduction”, “salicylic acid or jasmonic acid biosynthetic/metabolic processes” and “salicylic acid or jasmonic acid mediated signaling pathway” (**Supplementary Table 14**). Salicylic and jasmonic acid are plant hormones which can mediate defense responses against abiotic and biotic stresses (Browse, 2009; Kim et al., 2017). Therefore, the rapid expansion of stress-responsive gene families might underlie strong stress resistance of the *Rhododendron* species. To investigate potential WGD events in *Rhododendron* species, *Ks* was calculated for collinear paralogous genes in five *Rhododendron* species, as well as *A. chinensis* (kiwifruit) and *V. vinifera* (grape). The age distribution of *Ks* showed three peaks for kiwifruit, representing the three putative WGDs (Ad-α; Ad-β and At-γ), which supported the findings of previous reports (Huang et al., 2013; Wang et al., 2018; Wu et al., 2019). For the five *Rhododendron* species, two *Ks* peaks were observed, suggesting that the *Rhododendron* experienced only two rounds of polyploidy events (**Figure 2C**), of which the earliest event is shared by all core eudicots (γ triplication, WGT-γ). To further infer the polyploidy in *Rhododendron* species, we conducted collinearity and synteny analysis of *R. delavayi* genome (as a representative of *Rhododendron*) with grape and kiwifruit. Visualization of macrosynteny showed some ancestral regions in grape had two corresponding copy regions in *R. delavayi*, and that there were approximately two copies of each of these regions from *R. delavayi* in kiwifruit (**Supplementary Figure S6**). *Ks* distribution of collinear orthologs between kiwifruit (Actinidiaceae, *Actinidia*) and five studied *Rhododendron* plants revealed that *Rhododendron* and *Actinidia* diverged within the time frame of the Ad-β WGD in kiwifruit (**Figure 2D**). Previous studies suggested that *Actinidia* and *Rhododendron* shared the same WGD (Ad-β) that occurred near the Cretaceous-Tertiary (K-T) boundary (Wu et al., 2019). Here, we further explored whether the Actinidiaceae and Ericaceae shared the same β WGDs. The paralogous gene pairs of kiwifruit and *R. delavayi* (Ericaceae) from their β WGDs were extracted, respectively and orthologous relationships between them were established (**Supplementary Table 15**). Then, a phylogenetic tree was constructed for these genes, which revealed that the divergence of *R. delavayi* and kiwifriut paralogous genes was earlier than that of *R. delavayi–*kiwifriut orthologous genes (**Supplementary Figure S7**), implying that they shared the same Ad-β WGD, which is likely to have occurred before their lineages diverged. Consistent with previous research (Yang et al., 2020), the recent β WGD event in *Rhododendron* occurred ~78 Mya.

### Gene duplication contributes differently to gene family expansion

**Figure 3:**
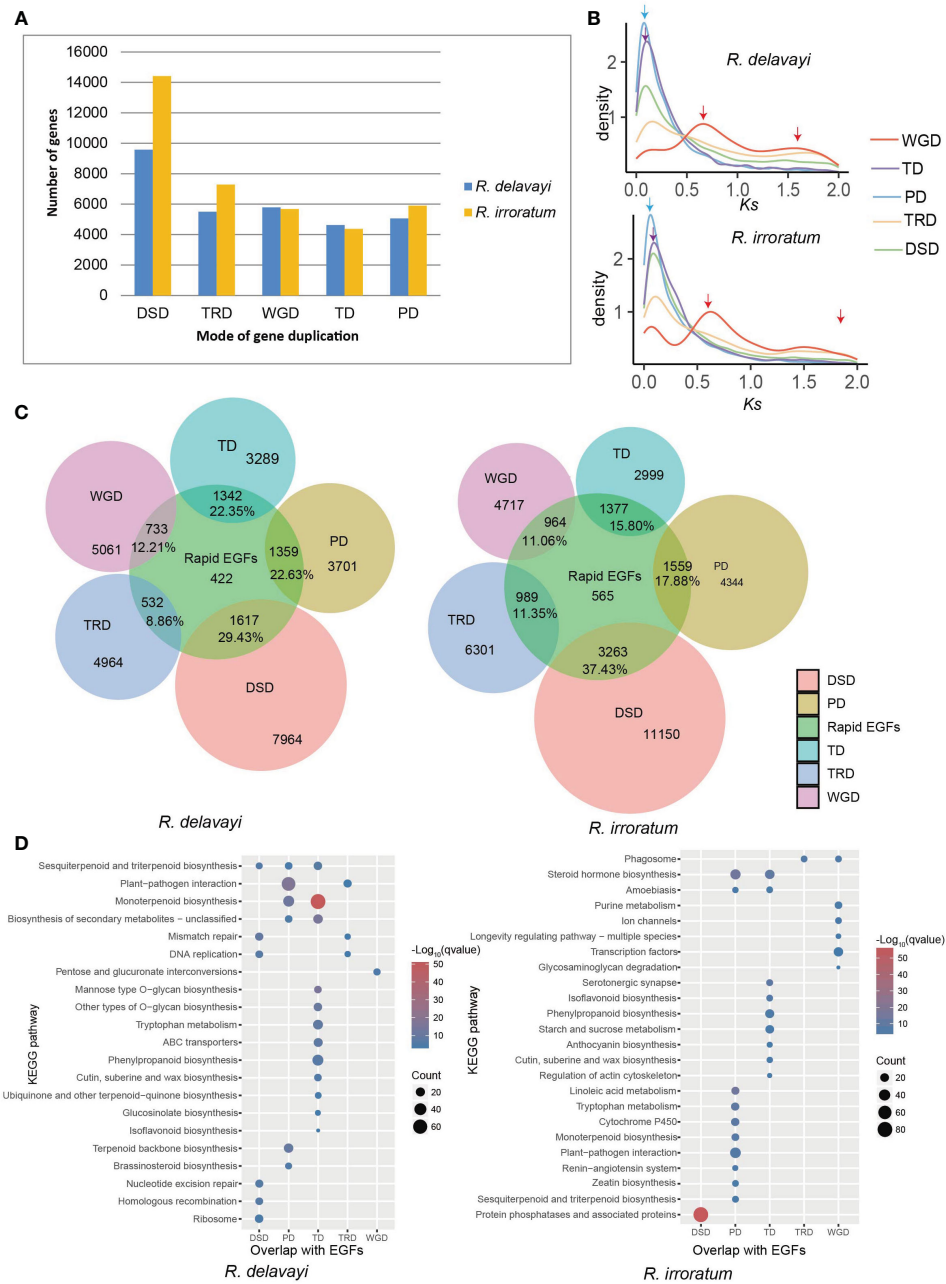
Gene duplication in *Rhododendron* and its contribution to gene family expansion. **(A)** Number of different kinds of duplicated genes in *R. delavayi* and *R. irrorarum*. WGD, whole-genome duplication; TD, tandem duplication; PD, proximal duplication; TRD, transposed duplication; DSD, dispersed duplication. **(B)** The *Ks* distributions of gene pairs derived from different modes of duplication. The red, purple, and blue arrows indicated the *Ks* peak of WGD-derived, TD-derived and PD-derived genes, respectively. **(C)** The relations between members of rapid expanded gene family (rapid EGFs) and members derived from different modes of duplication in *R. delavayi* and *R. irroratum*. **(D)** KEGG enrichment of genes in rapid EGFs and the different modes by which these genes have duplicated. The color and size of the circles represent statistical significance and the number of genes in a KEGG pathway, respectively; ‘qvalue’ is the correted P value.

Gene duplication has been considered as a key driving force of evolutionary novelties in plants (Flagel and Wendel, 2009; Panchy et al., 2016). Duplicated genes can be categorized into five types according to their origins (Qiao et al., 2019): whole-genome duplication (WGD), tandem duplication (TD), transposed duplication (TRD), proximal duplication (PD) and dispersed duplication (DSD). In addition to WGD, other patterns of duplications are always regarded as single-gene duplications (Qiao et al., 2019). Here, we identified 30,562 and 37,653 duplicated genes in *R. delavayi* and *R. irroratum* genomes, respectively and classified each as belonging to one of the five categories (**Figure 3A**; **Supplementary Table 16**). In both species, the DSD-derived genes accounted for the largest proportion. Two *Ks* peaks were found for gene pairs originating from WGDs, representing the two WGD events occurred in *Rhododendron*. Notably, gene pairs derived from TD and PD events exhibited only one *Ks* peak with lower *Ks* values that occurred after the WGD-β event, indicating the TD and PD duplications burst more recently (**Figure 3B**).

We analyzed the contribution of different duplicates to gene family expansion by searching for overlaps between each category of gene duplications and the rapid EGFs (**Figure 3C**). Of all the duplication types, DSD contributed the largest proportion to gene family expansion, followed by TD and PD, in both *R. delavayi* and *R. irroratum*. The KEGG enrichment analysis for genes overlapping between rapid EGFs and WGD-TD-PD-TRD-DSD indicated that different duplication-induced gene family expansions were enriched in divergent KEGG pathways (**Figure 3D**). For instance, the expansion of gene families relevant to “phenylpropanoid”, “isoflavonoid” and “cutin, suberine and wax” biosynthesis were largely caused by TD, and expansion of “plant-pathogen interaction” genes was primarily attributed to PD events in both species. Moreover, in *R. delavayi*, the expansion of gene family participating in “terpenoid backbone biosynthesis” predominantly resulted from PD events, while the gene family expansions of “sesquiterpenoid and triterpenoid” and “monoterpenoid” biosynthesis were mainly induced by TD and PD events. In comparison, in *R. irroratum*, PD mainly contributed to the gene expansion of “monoterpenoid”, “sesquiterpenoid and triterpenoid” biosynthesis and the expansion of “anthocyanin biosynthesis” gene families was largely due to TD events. WGD events largely accounted for the expansion of gene families involved in basic biological functions or transcription factors, e.g. pentose and glucuronate interconversions in *R. delavayi* and purine metabolism, ion channels, transcription factors in *R. irroratum*. It is observed that the DSD events mainly contributed to expansion of gene families relevant to DNA replication, repair and homologous recombination in *R. delavayi*. Taken together, the contributions of different duplication modes to gene family expansion were different and the newly generated TD/PD might be important sources of expansion for gene families related to secondary metabolite biosynthesis and pathogen resistance. The flavonoids, terpenoids (isoprenoids) and ‘cutin, suberine and wax’ are substantially characterized as defense metabolites (barriers) because of their critical roles in plant abiotic or biotic stresses (Nakabayashi and Saito, 2015; Lepiniec et al., 2006; Pu et al., 2021; Lee and Suh, 2015). Thus, TD/PD duplications would enhance the ability of stress tolerance to various environmental stresses in the investigated species.

### Evolution and comparative investigation of the R2R3-MYB subfamily

**Figure 4:**
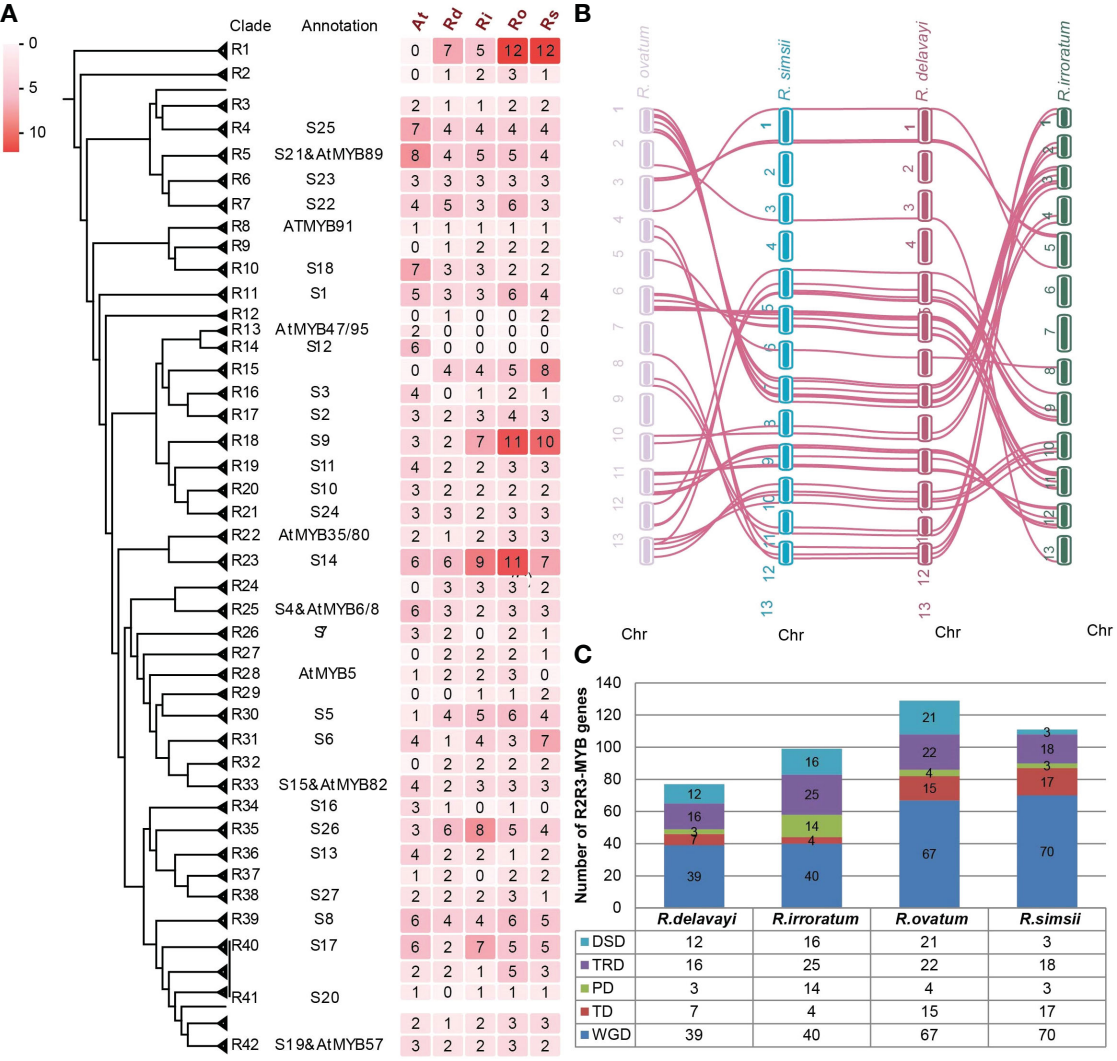
Comparative evolutionary analysis of the R2R3-MYB subfamily members in *Rhododendron*. **(A)** Phylogenetic tree of R2R3-MYB members from four *Rhododendron* species and *Arabidopsis*. The folding triangles represented the 42 defined subgroups (R1 to R42). One protein from *R. delavayi* (line) did not fit well into subgroups. Subgroups clustered with Arabidopsis R2R3-MYB proteins were annotated with designated *A thaliana* subgroups as previously reported (Dubos et al., 2010). Number of subgroup members for each species was indicated by the heatmap. The uncompressed tree was shown in **Supplementary Figure S13**. **(B)** Interspecies synteny of *R2R3-MYB* genes among four *Rhododendron* genomes; **(C)** Number of *R2R3-MYB* genes produced by distinct gene duplications.

We searched the proteomes of four studied *Rhododendron* species to identify candidate MYB proteins using the HMM method. The proteins that contained two MYB DNA-binding repeats (Rs) were considered as the R2R3-MYB subfamily proteins. Finally, 102, 118, 151 and 133 *R2R3-MYB* genes were obtained for *R. delavayi*, *R. irroratum*, *R. ovatum*, *R. simsii*, respectively. Based on the genome annotations, 102 *R. delavayi*, 113 *R. irroratum*, 151 *R. ovatum* and 125 *R. simsii R2R3-MYB* genes were located on 13 chromosomes, respectively, which showed uneven distributions in each of the four *Rhododendron* species (**Supplementary Figure S8**). For example, chromosome 8 had the largest number of genes (14), while, chromosome 2 had only one gene in *R. delavayi*. The total number of *R2R3-MYB* genes of *R. delavayi* and *R. irroratum* was less than that of *R. ovatum* and *R. simsii*. To figure out the relatively small number of *R2R3-MYB* genes in *R. delavayi* and *R. irroratum* was due to gene loss or failure of gene annotations, CDS sequences of *R. irroratum* R2R3-MYBs were used as query to search the *R. delavayi* genome sequence with BLAT v.36x2 and vice versa. Finally, only 11 and 10 matched genomic regions were not structurally annotated in the *R. delavayi* and *R. irroratum* genomes, respectively. Therefore, the lower number of *R2R3-MYB* genes in *R. delavayi* and *R. irroratum* was likely caused by gene loss, pseudogenization and failure of annotation. To understand their group classification and evolutionary relationship, a phylogenetic tree was constructed using all R2R3-MYB proteins of *Arabidopsis* and four *Rhododendron* species. The R2R3-MYB members were separated into 42 subgroups (designed as R1 to R42) (**Figure 4A**; **Supplementary Figure S9**) based on the tree and subgroup classification of R2R3-MYB proteins of *Arabidopsis*. Compared to *Arabidopsis*, the R2R3-MYB members in 9 and 13 subgroups exhibited expansion and contraction for all four *Rhododendron* species, respectively. Specifically, the 9 expanded subgroups (R1, R2, R9, R12, R15, R24, R27, R29 and R32) consisted of *Rhododendron* specific members, implying their possible distinctive roles in *Rhododendron*. The *R. delavayi* gene *Rhdel07G0021400* was not categorized into any subgroup. The exon/intron patterns and conserved protein motifs of the *R2R3-MYB* genes suggested that most members in each subgroup were usually characterized by similar exon/intron patterns and motif distributions, whereas members belonging to different subgroups might have different exon/intron organizations and motif characteristics, e.g., the members belonging to subgroup R1 had two exons and one intron, while members of subgroup R3 had more than six exons and six introns (**Supplementary Figure S9**). Therefore, the gene structures and protein motif composition showed relatively higher conservation for *R2R3-MYB* genes of the same subgroup, which might be conserved in evolution. Considering that gene family members clustered together in the phylogeny usually tended to possess similar biological functions or control the identical metabolic pathway, it is possibility to perform function prediction of the *R2R3-MYB* genes in *Rhododendron*, based on their phylogenetic relationships with the *Arabidopsis* orthologs, of which the functions have been well studied. It has been reported that *AtMYB75/PAP1*, *AtMYB90/PAP2*, *AtMYB113* and *AtMYB114* (subgroup 6) were involved in the regulation of anthocyanin biosynthesis in *Arabidopsis* (Gonzalez et al., 2008). On the phylogenetic tree, these four genes were clustered with 15 *Rhododendron R2R3-MYB* genes, including one in *R. delavayi* (*Rhdel08G0093800*), four in *R. irroratum (Ri_282360*, *Ri_185830*, *Ri_282290*, *Ri_282390)*, three in *R. ovatum* (Ro_22285, Ro_22282, Ro_22319) and four in *R. simsii* (*Rhsim08G0087300*, *Rhsim08G0087200*, *Rhsim08G0087400*, *Rhsim10G0209900*, *Rhsim08G0087500*, *Rhsim08G0087600*, *Rhsim08G0087800*), which can be important candidate genes for exploration of their functions in anthocyanin synthesis and flower coloration in *Rhododendron* species. In addition to plant secondary metabolism, the *R2R3-MYB* proteins also participate in stress responses. For example, *Arabidopsis R2R3-MYB* genes such as *AtMYB60*, and *AtMYB96* (subgroup 1) have been proven to participate in drought response through mediating stomatal movement (Cominelli et al., 2005) and abscisic acid signaling pathway (Seo et al., 2009). In addition, *AtMYB96* can also be involved in ABA-mediated pathogen resistance (Seo and Park, 2010). These genes were clustered with 16 *Rhododendron R2R3-MYB* genes, including three in *R. delavayi* (*Rhdel10G0043900*, *Rhdel12G0197900*, *Rhdel08G0197900*), three in *R. irroratum* (*Ri_139470*, *Ri_195160*, *Ri_293310*), six in *R. ovatum* (*Ro_35465*, *Ro_09930*, *Ro_29255*, *Ro_14416*, *Ro_06615*, *Ro_31578*) and four in *R. simsii* (*Rhsim10G0039500*, Rhsim03G0045800, *Rhsim12G0156400*, *Rhsim08G0155100*), which can be further studied to illuminate their functions in stress resistance in *Rhododendron*.

The interspecific synteny of the *R2R3-MYB* genes among four *Rhododendron* species was investigated to explore their evolutionary conservation. First, the R2R3-MYB genes in syntenic blocks between any two genomes were determined based on the MCScanX analysis and 41 one-to-one orthologous genes among the four species were selected (**Figure 4B**; **Supplementary Table 17**), suggesting their conserved characteristics during the evolution. The duplicated mechanisms that generated the *R2R3-MYB* subfamily were also analyzed and results showed that WGD events contributed to more than 50% of the overall gene number in each of the four R*hododendron* plants (but not in *R. irroratum*) (**Figure 4C**).

### Evolution of the NBS-encoding genes

**Figure 5:**
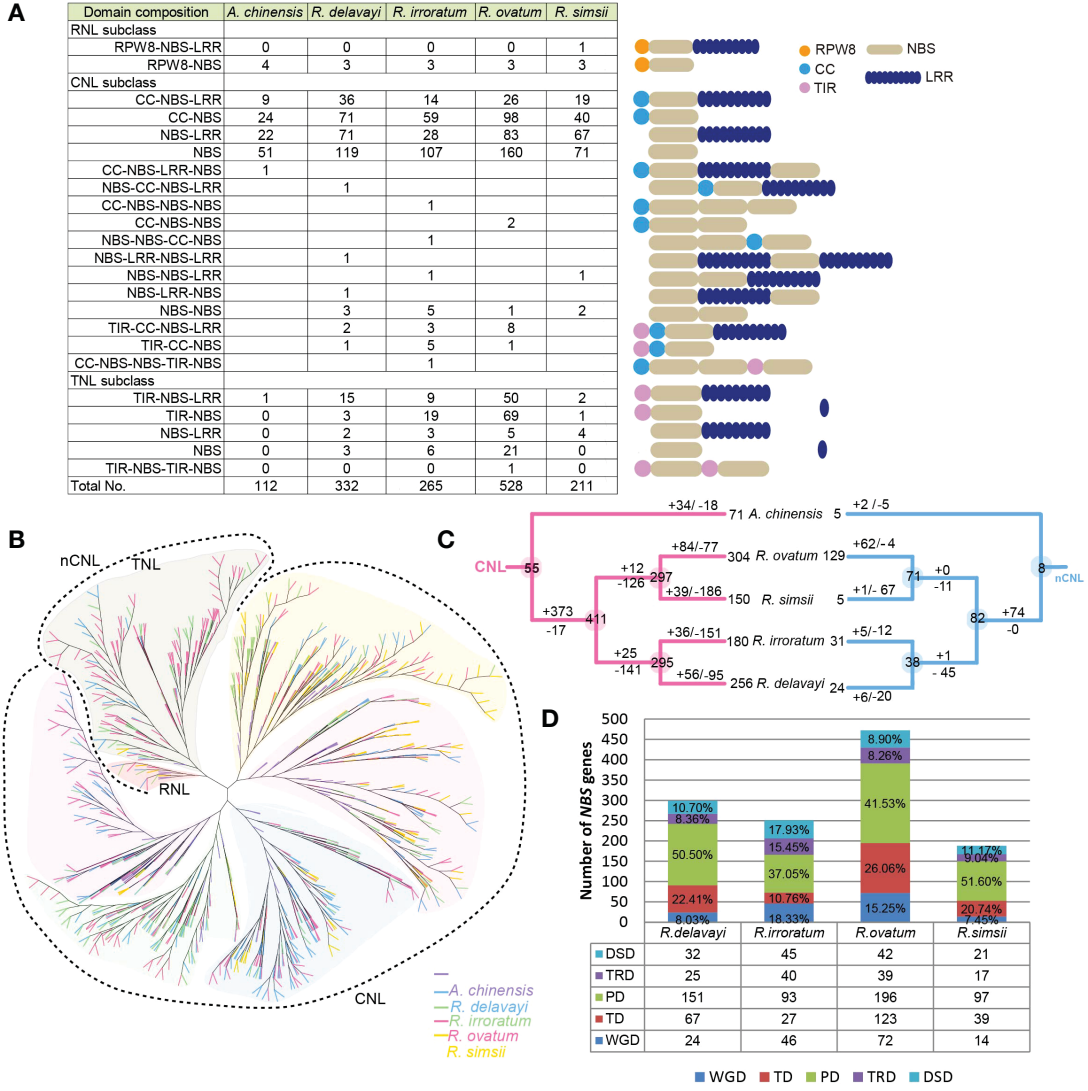
NBS-encoding gene evolution in *Rhododendron*. **(A)** Number of the NBS-encoding genes in each category in four *Rhododendron* species and kiwifruit. Twenty-three domain combinations were found. The numbers listed in table represented the number of genes for each domain combination. ‘R’, ‘C’, ‘T’ and ‘N’ were the abbreviations of RPW8, CC, TIR and NBS domains. Genes with both CC and TIR domains were clustered into CNL clade based on phylogenetic analysis. **(B)** The NBS-encoding gene phylogenetic tree for four *Rhododendron* species and kiwifruit; **(C)** Duplications/losses of the NBS-encoding genes during the evolutionary processes of *Rhododendron*. The number of ancestral genes for each node was shown in circles. Gene duplications or losses on each branch were illustrated by numbers with + or –symbols, respectively; **(D)** Numbers of NBS-encoding genes produced by different gene duplication events in four *Rhododendron* genomes.

In this study, the NBS-encoding genes were identified and their evolutionary patterns were investigated during the speciation of four *Rhododendron* species. Using the HMM method, a total of 1,336 candidate genes were identified in four *Rhododendron* species: 332 from *R. delavayi*, 265 from *R. irroratum*, 527 from *R. ovatum*, and 211 from *R. simsii*. The NBS-encoding genes presented prominent expansion in *Rhododendron* genomes, compared with kiwifruit (102 genes). According to their N-terminal domains, these obtained genes were first classified into three subclasses: CNL, TNL and RNL. For each subclass, the genes were further categorized into different types based on their predicted protein domain compositions (**Figure 5A**). In addition, sequences that lacked the TIR, CC, or RPW8 domains were subjected to BLASTn analyses with the classified genes to determine their subclass classification based on sequence similarity. Cluster assignment suggested the majority of NBS-encoding genes were located in clusters on *Rhododendron* chromosomes and only a small number of genes existed as singletons (**Supplementary Table 18**). The domain compositions and cluster assignment of the 1,336 genes were shown in **Supplementary Table 19**.

An overall phylogeny was constructed for NBS-encoding genes from four *Rhododendron* species as well as kiwifruit, which revealed that the RNL clade was embedded in the TNL clade, and CNL formed an independent clade (**Figure 5B**). This phylogenetic tree was inconsistent with the topology in previous studies, in which the three subclasses (TNL, CNL and RNL) each formed a monophyletic clade (Shao et al., 2016). Here, two phylogenies for NBS-encoding genes (CNL and nCNL) were reconstructed and reconciled with the real species tree to restore ancestral gene duplication or loss events (**Supplementary Figure S10**, **S11**). According to the definitions, 63 ancient NBS-encoding gene lineages (55 CNL, 8 nCNL) were recovered in the common ancestor of kiwifruit and four *Rhododendron* species (**Supplementary Tables 20, 21**). Of these ancient gene lineages, 46 lineages (38 CNL, 8 nCNL) were inherited by the common ancestor of four *Rhododendron* species and further diverged into 493 gene lineages (411 CNL, 82 nCNL), which were defined as *Rhododendron* NBS-encoding gene lineages. Since divergence from kiwifruit, the 493 ancestral *Rhododendron* NBS-encoding gene lineages underwent different gene duplication/loss events during the speciation of four *Rhododendron* species. It is found that 141 CNL lineages and 45 nCNL lineages were lost in the common ancestor of *R. delavayi* and *R. irroratum*, while 25 CNL and 1 nCNL lineages were gained, resulting in 295 CNL and and 38 nCNL lineages (**Figure 5C**). Then, the gene duplications occurred less frequently than gene losses in both *R. delavayi* and *R. irroratum*, causing a more reduced number of genes in the two species. Meanwhile, 126 CNL and 11 nCNL lineages were lost, while 12 CNL and 0 nCNL lineages were gained in the common ancestor of *R. simsii* and *R. ovatum*, resulting in 297 CNL genes and 71 nCNL genes (**Figure 5C**. However, *R. simsii* and *R. ovatum* underwent considerably different evolutionary processes after divergence. Specifically, *R. ovatum* duplicated 146 genes (84 CNL, 62 nCNL), and lost 82 genes (77 CNL, 4 nCNL), resulting in an increased number of NBS-encoding genes, especially for nCNL subclass. *R. simsii* duplicated 40 genes (39 CNL, 1 nCNL) and lost 253 genes (186 CNL, 67 nCNL), resulting in a decreased gene number in this genome. In general, the NBS-encoding genes appeared to experience abundant duplications in the common ancestor of four *Rhododendron* species. Then, more frequent gene losses than gains occurred during the speciation of *R. delavayi*, *R. irroratum*, and *R. simsii*. However, *R. ovatum* was the opposite.

The NBS gene duplications revealed that PD and TD events were responsible for larger proportion of the NBS-encoding genes in *R. delavayi* (73.91%), *R. ovatum* (67.58%) and *R. simsii* (72.34) genomes, whereas PD and WGD events produced more than of the genes (55.38%) in *R. irroratum* (**Figure 5D**). In particular, the PD-derived NBS-encoding genes occupied the largest proportion across all studied *Rhododendron* species (**Figure 5D**).

## Discussion

In this study, the chromosome-level genome assemblies were generated for two alpine rhododendrons, *R. delavayi* and *R. irroratum*, which can be used as the parent to produce hybrid varieties for ornamental or landscape use. The newly assembled *R. delavayi* genome had higher accuracy and continuity than the previously reported version (Zhang et al., 2017). In view of the relatively high heterozygosity (1.55%) of *R. irroratum* genome, Purge Haplotigs program were used to remove duplicated haploid contigs from the contig-level assembly. Through Hi-C scaffolding and manual correction, the genome sequences of *R. delavayi* and *R. irroratum* we can assemble into 13 pseudo-chromosomes with contigs/scaffolds accurately assigned, in accordance with the chromosome number mentioned by previous study (Zhang et al., 2007). Phylogenetic analysis placed five *Rhododendron* species into two sister clades, with that comprising *R. delavayi*, *R. irroratum* and *R. williamsianum* referred to as ‘rhododendrons’ and the other clade, containing *R. simsii* and *R. ovatum*, commonly considered as the ‘azaleas’ (De Riek et al., 2018). We dated a WGD event (Ad-β) in *Rhododendron* at around 78 Mya, prior to the divergence of *Rhododendron* and *Actinidia.* Although the *Rhododendron* genus contains the most diverse species of woody plants, there was no evidence for the occurrence of a recent WGD in the common ancestor of *Rhododendron* (Ericaceae). A study based on phylogenomic and ecological analyses shed light on the evolutionary radiations and species diversity of *Rhododendron* are driven by geographic, climatic factors and functional traits of leaves (Xia et al., 2022).

Gene duplications can provide important raw materials for evolutionary novelty and the duplicated genes were often biased retained for different modes of duplication (Freeling, 2009). Our analysis revealed that the duplication-involved expanded gene families were enriched in different KEGG pathways. TD/PD duplications substantially contributed to the expansion of gene families related to secondary metabolism and plant-pathogen interaction, while genes involved in basic biological process tended to be expanded by WGD event. The gene retentions after WGD versus TD/PD tend to be biased and might exhibit reciprocal relationship, which can be explained by balanced gene drive (Freeling, 2009). Different from whole-genome duplication, the tandem or proximal duplicates can emerge continuously (Qiao et al., 2019), to some extent, which can increase gene dosage efficiently (Conant and Wolfe, 2008) and play significant role in rapid environmental response (such as pathogen invasion) or rate-limiting steps in secondary metabolism.

The R2R3-MYB proteins have been proven to control various biological processes in plants, e.g. secondary metabolism, development and stresses responses (Dubos et al., 2010). Here, the R2R3-MYB members were comprehensively identified and characterized in four *Rhododendron* species. The number of *R2R3-MYB* genes in *R. ovatum* and *R. simsii* is more than that in *R. delavayi* and *R. irroratum*, which might be attributed to frequent gene losses, pseudogenization or failure of gene annotations. It was noteworthy that these *Rhododendron* genomes were annotated using different strategies, and there must be incomplete or error annotation to some extent. Thus, it is necessary to annotate these *Rhododendron* genomes in a uniform way. There were only 41 one-to-one syntenic orthologs found in the four *Rhododendron* species. The remaining genes either maintain collinearity relationships in two or three *Rhododendron* genomes or derived from other duplicated modes, such as tandem duplication, transposed duplication and so on. In addition, genes in the syntenic blocks might also experience loss or translocation.

Flower color is a critical ornamental characteristic of *Rhododendron* and is mainly determined by anthocyanins and carotenoids (Yang et al., 2020). Studies have demonstrated that the R2R3-MYB transcription factors are key regulators in the biosynthesis of anthocyanin and carotenoid in plants (Schwinn et al., 2006; Albert et al., 2014; Sagawa et al., 2016; Ampomah-Dwamena et al., 2019). Since the R2R3-MYB members within subgroups are conservative and functionally correlated, 15 *Rhododendron R2R3-MYB* genes clustered together with *Arabidopsis AtMYB75/AtMYB90/AtMYB113/AtMYB114* genes were supposed to be related to anthocyanin biosynthesis. These genes could be important candidates to further investigate their roles in flower coloration of *Rhododendron*. Moreover, we identified 16 *Rhododendron R2R3-MYB* genes clustered together with Arabidopsis MYB genes *AtMYB60* and AtMYB96, which might participate in drought stress response. Gene duplication is critical in gene family expansion and evolution. Here, the contributions of different gene duplications to *R2R3-MYB* gene expansions were dissected and the results revealed that WGD event played predominant roles in generating the *R2R3-MYB* genes for four investigated *Rhododendron* species, agreeing with previous findings that genes encoding transcription factors are preferentially retained after polyploidization (Tian et al., 2005).

Plants are threatened by various pathogens in their natural habitats. We found several gene families participating in biotic stimuli and defense responses such as “plant-pathogen interaction” and “immune response-activating signal transduction” were significantly enriched. It should be noted that NBS-encoding genes play crucial roles in plant disease resistance and immune response. Therefore, we identified this type of R genes and found they presented obvious expansion in *Rhododendron* genus compared with kiwifruit, suggesting the wild *Rhododendron* species may have strong resistance to disease erosion. *R. ovatum* had more identified NBS-encoding genes than other three *Rhododendron* species, perhaps because it is distributed in the subtropical zone at lower altitudes and in hotter environments where the plant may be more exposed to microorganisms. There were 493 ancestral *Rhododendron* NBS-encoding gene lineages, from which the genes detected currently in *Rhododendron* species should be derived. After divergence from common ancestor, the individual *Rhododendron* species experienced different numbers of gene gain/loss: *R. delavayi*, *R. irroratum* and *R. simsii* exhibited a “continuous contraction” pattern (for both CNLs and nCNLs), because these species lost more genes than they gained at two periods (before and after the split of the common ancestors of *R. delavayi*, and *R. irroratum*, and of *R. simsii* and *R. ovatum*) and *R. ovatum* showed a “contraction followed by expansion” pattern (for both CNLs and nCNLs) by losing more genes before the split with *R. simsii* but subsequently gaining more genes. Therefore, the different gene gains and losses resulted in different number of NBS-encoding genes in the current *Rhododendron* genomes. Consistent with the above findings that TD/PD duplications play critical roles in the expansion of gene families involved in plant-pathogen interaction, the TD/PD events largely contributed to the expansion of NBS-encoding genes, suggesting these duplicates are important for plant response to pathogenic organisms.

In summary, the two newly available *Rhododendron* genome sequences add to the increasing available genome information for *Rhododendron* and will facilitate the study on genome evolution of Ericaceae. The candidate *R2R3-MYB* genes predicted to be involved in anthocyanin biosynthesis and stress response will provide potential targets for further functional characterization, which can guide breeders to develop new ornamental varieties and enhance stress response of *Rhododendron* cultivars. Moreover, the comprehensive identification and evolutionary analysis of NBS-encoding genes could provide a reference for further investigation of this gene family in *Rhododendron* species.

## Data availability statement

The raw sequence reads of R. delavayi/R. irroratum have been deposited in the NCBI under BioProject number PRJNA929713/ PRJNA931779 and in the CNCB under BioProject number PRJCA011631/PRJCA011815, respectively. Genome assembly and annotation files are available at the Rhododendron Plant Genome Database (http://bioinfor.kib.ac.cn/RPGD/download_genome.html).

## Author contributions

XWu, LZ, and RZ conducted the data analysis. XW, GJ and ZW assisted in the data analysis. HY and YH prepared the samples. JW, YM, CZ and XWu. wrote and revised the manuscript. All authors contributed to the article and approved the submitted version.

## Footnotes

1
https://github.com/ben-lerch/IsoSeq-3.0

2
https://github.com/Nextomics/NextDenovo

3
https://github.com/ruanjue/wtdbg

4
http://transdecoder.github.io

5
https://github.com/apetkau/orthomcl-pipeline

6
https://github.com/tanghaibao/jcvi/wiki/MCscan-(Python-version)

7
https://pfam.xfam.org/

8
http://plantregmap.gao-lab.org/

9
http://smart.embl-heidelberg.de/

10
https://www.ncbi.nlm.nih.gov/Structure/cdd/wrpsb.cgi

11
http://www.arabidopsis.org/
