# Supplementary figures and images for: Evolutionary history of two evergreen Rhododendron species as revealed by chromosome-level genome assembly
Source: Front Plant Sci. 2023 Mar 21;14:1123707. doi: 10.3389/fpls.2023.1123707 (PMC10070854; doi:10.3389/fpls.2023.1123707)

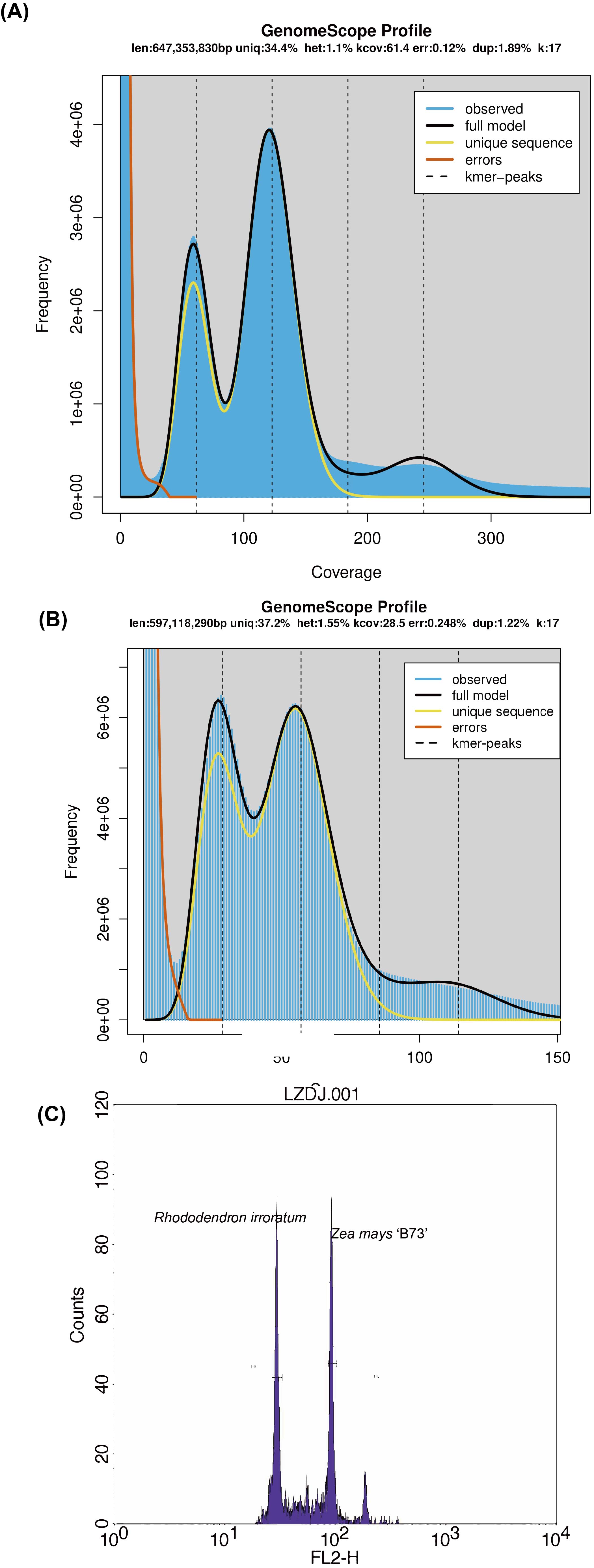

Supplement: Supplementary file 1 [file Image_1.jpeg]

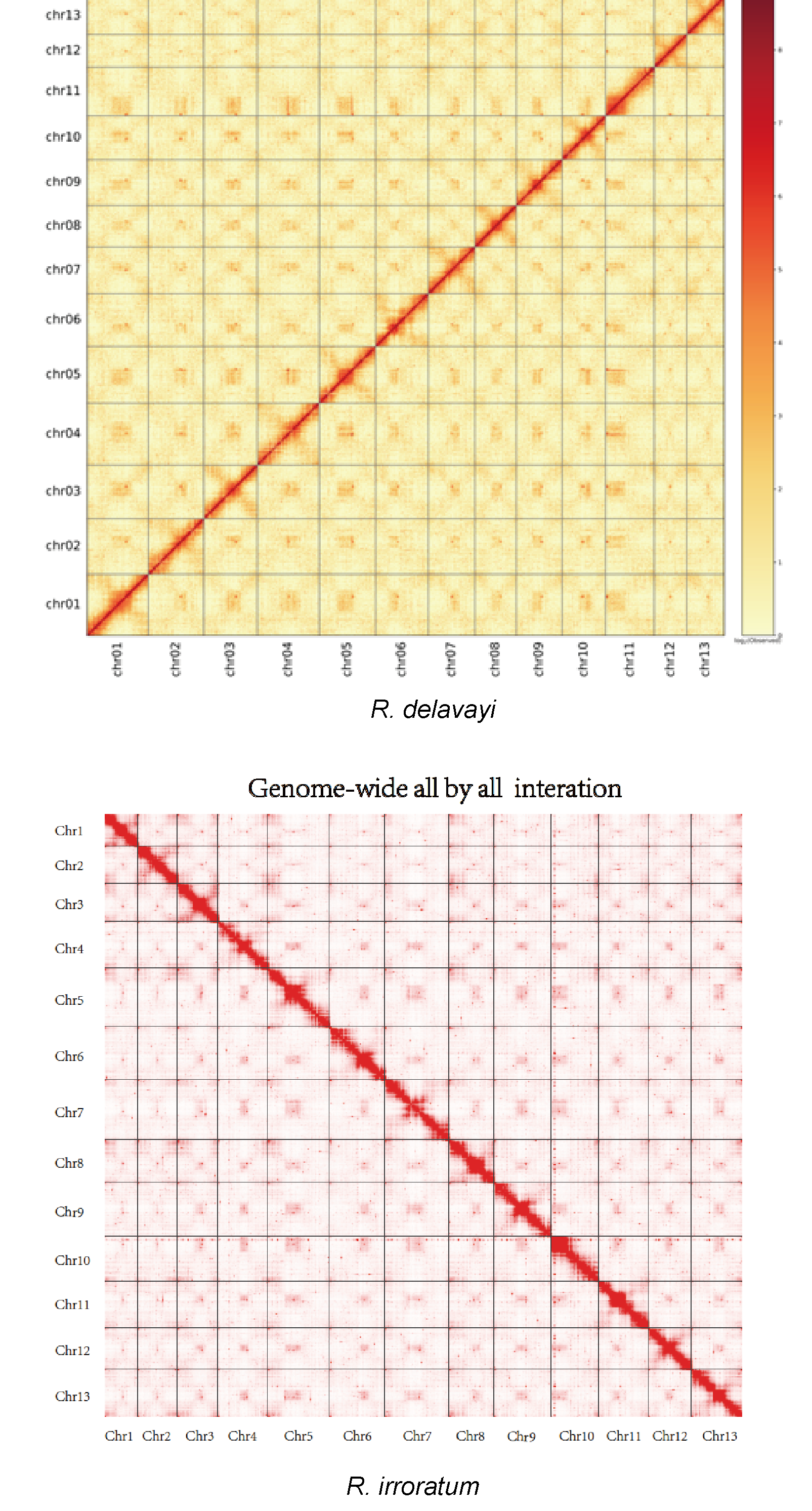

Supplement: Supplementary file 2 [file Image_2.tif]

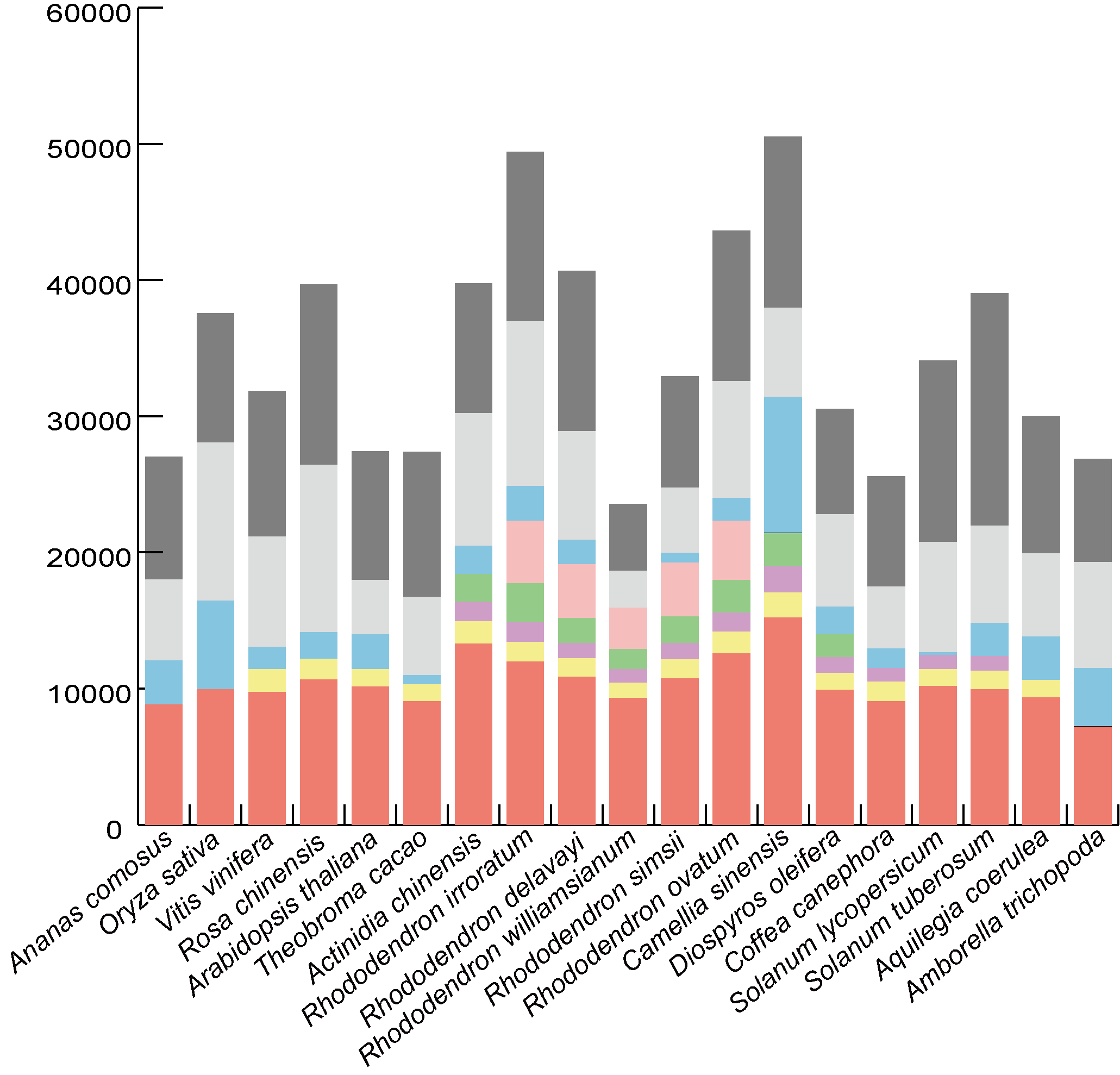

Supplement: Supplementary file 3 [file Image_3.tif]

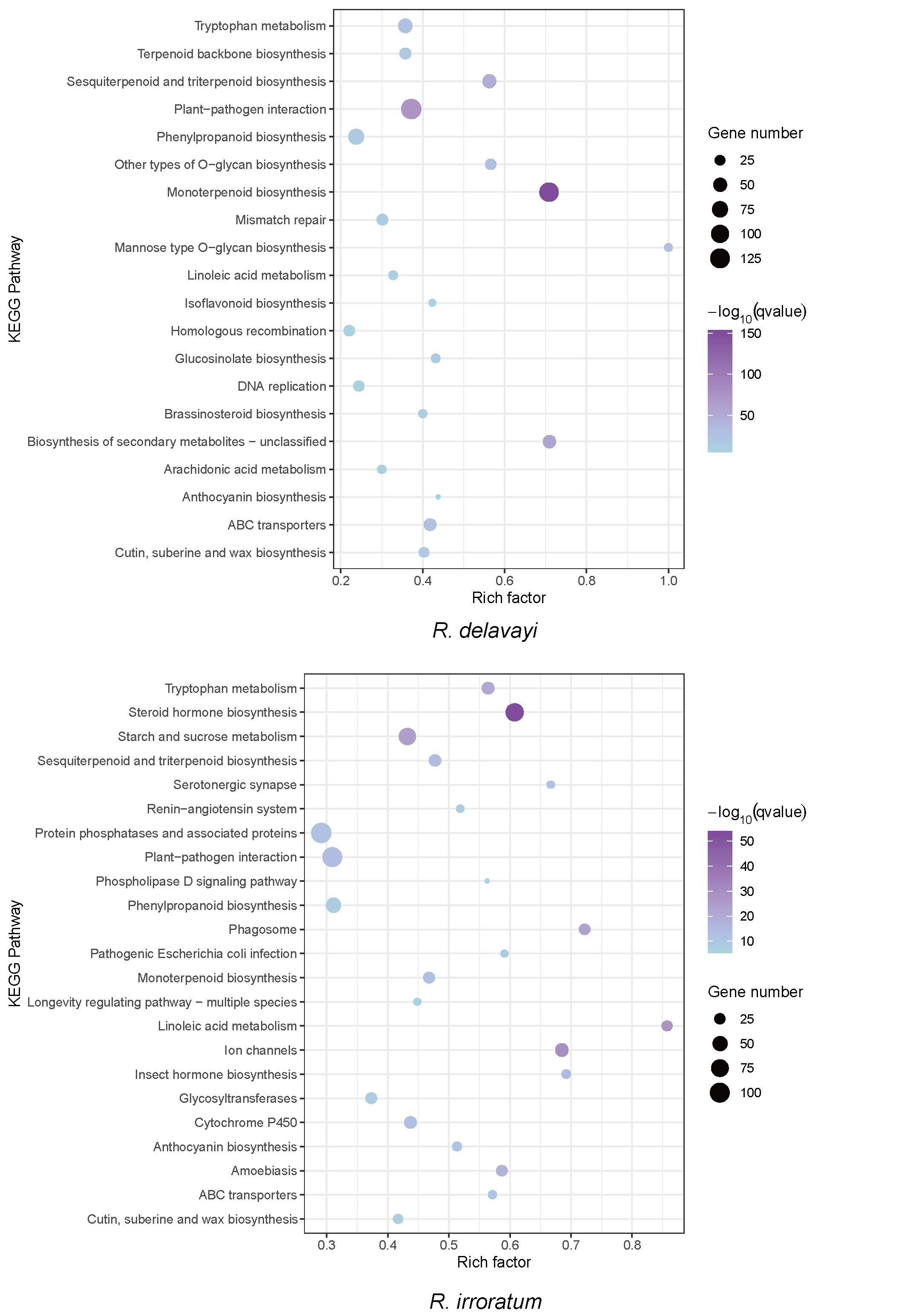

Supplement: Supplementary file 4 [file Image_4.tif]

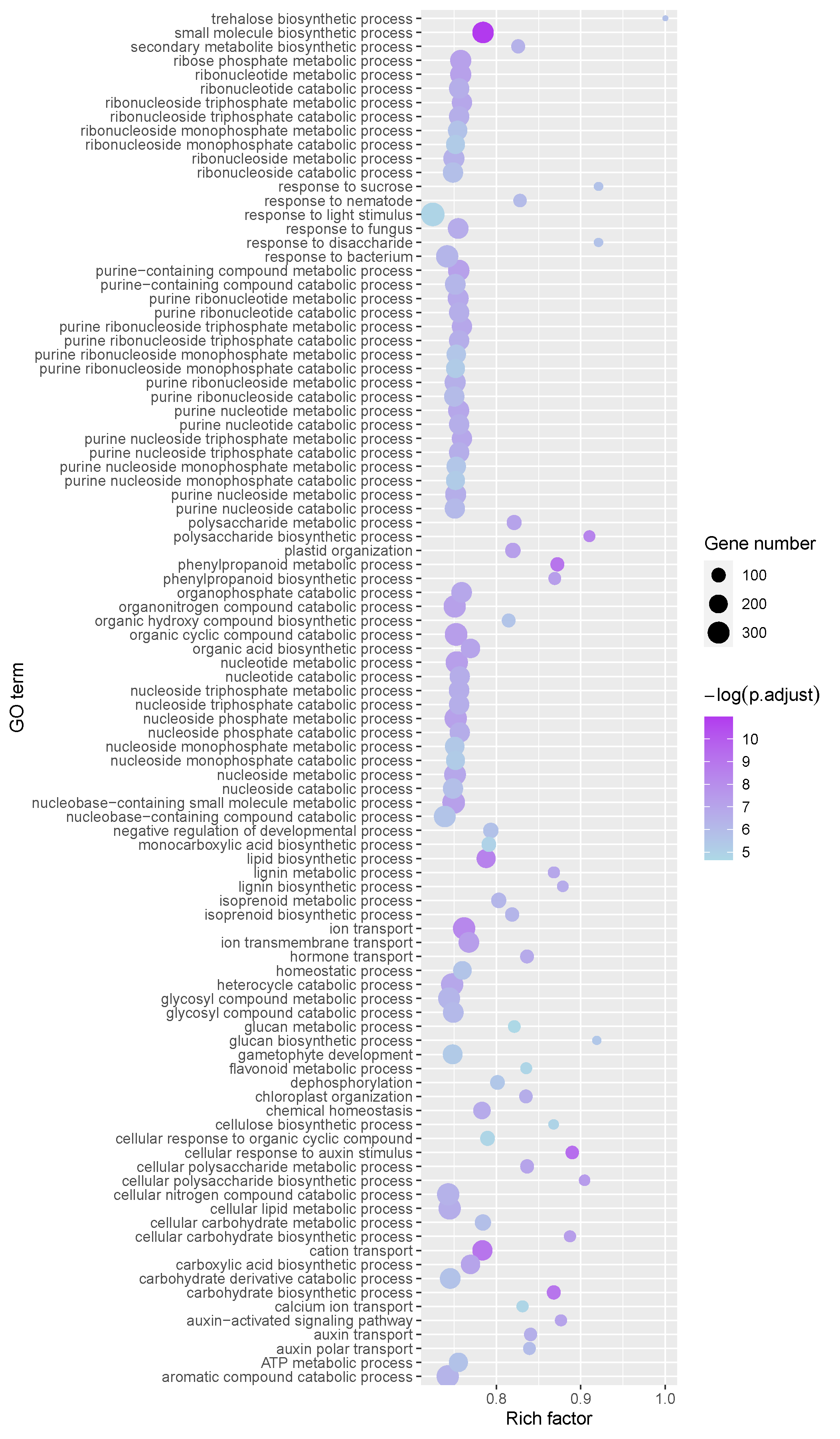

Supplement: Supplementary file 5 [file Image_5.tif]

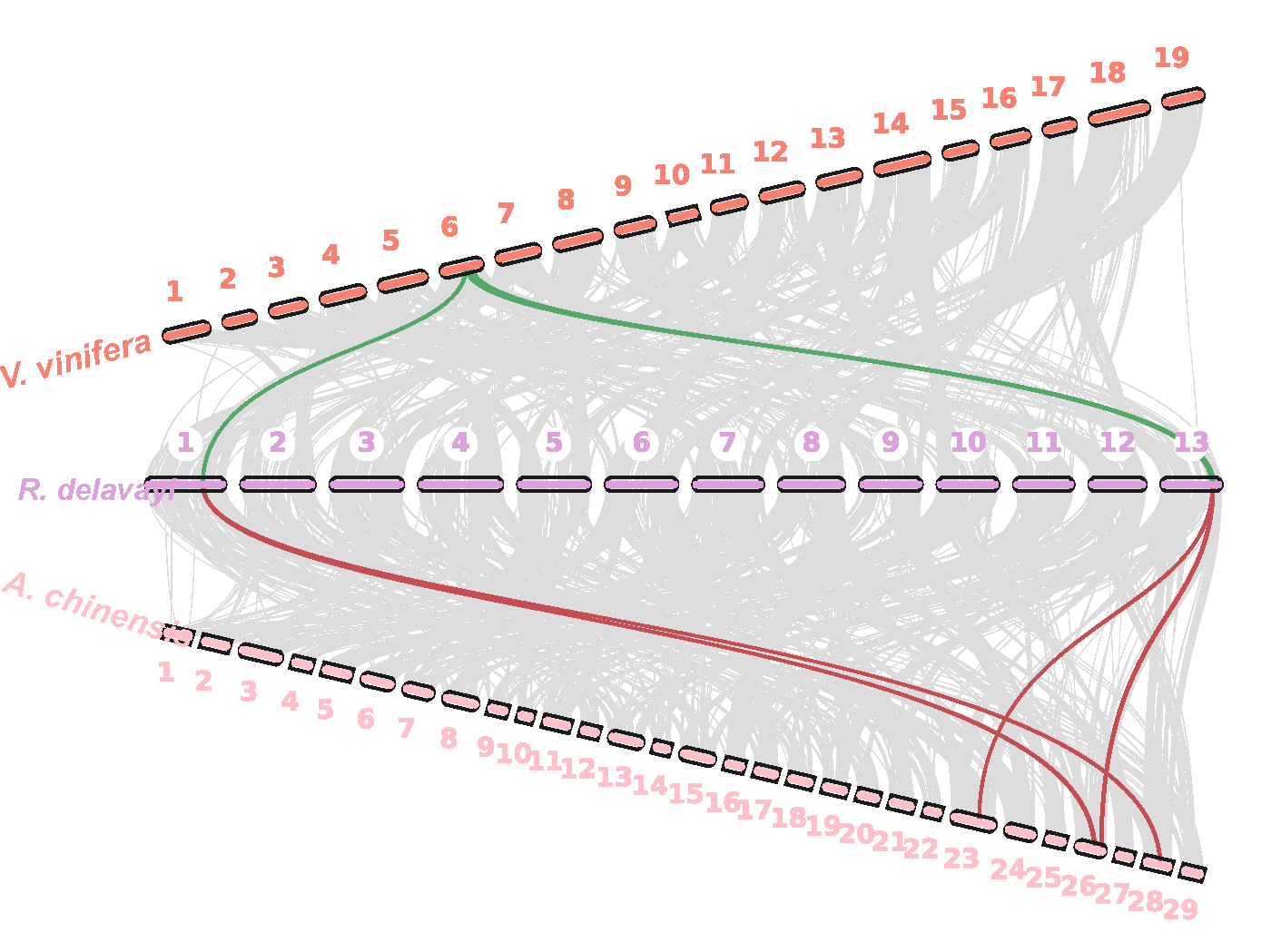

Supplement: Supplementary file 6 [file Image_6.tif]

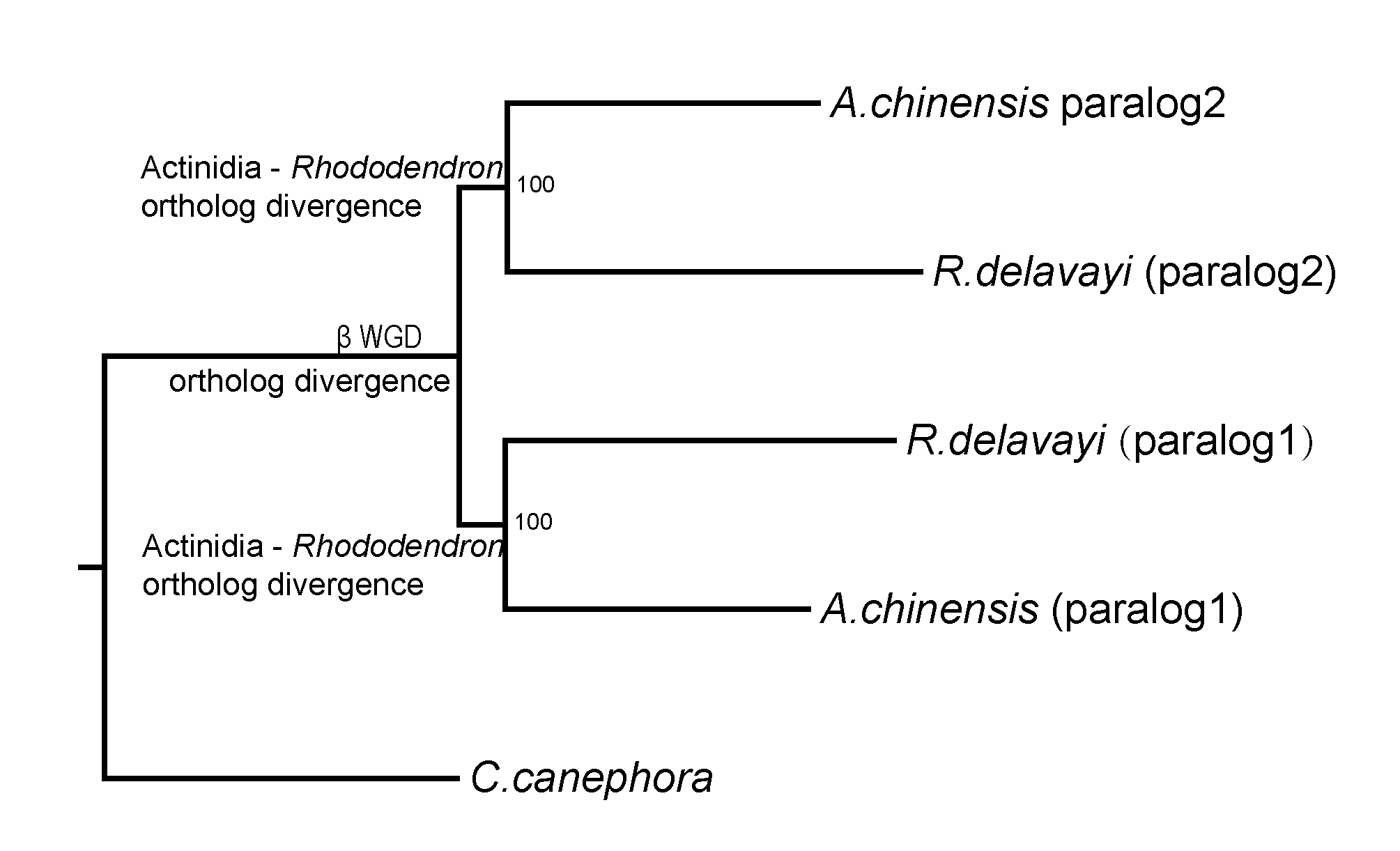

Supplement: Supplementary file 7 [file Image_7.tif]

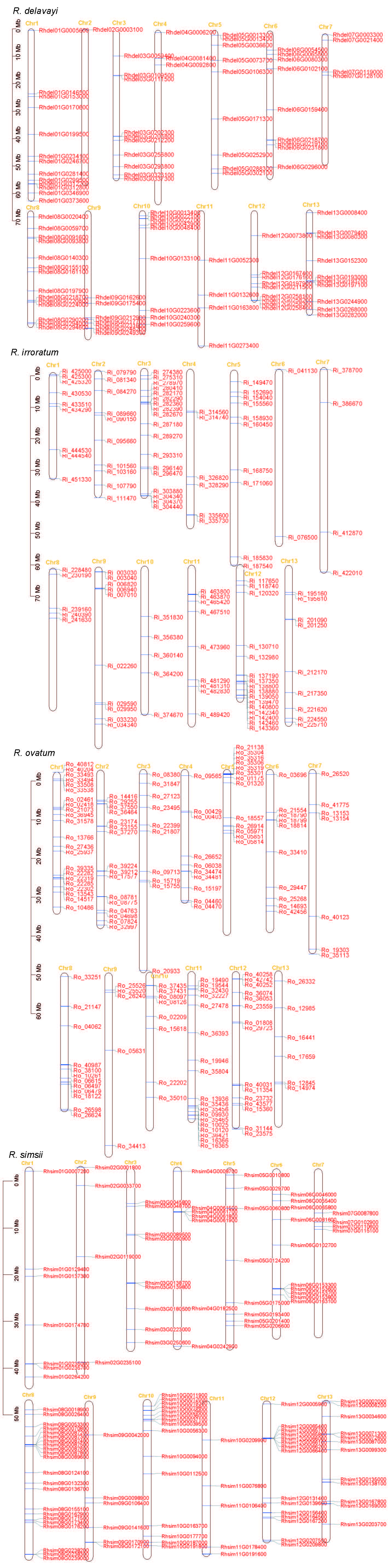

Supplement: Supplementary file 8 [file Image_8.jpeg]

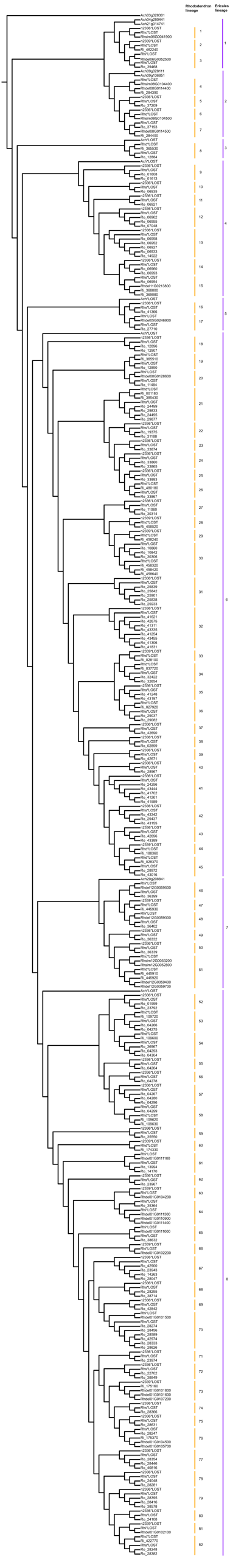

Supplement: Supplementary file 11 [file Image_11.tif]
